# Synthesis of Nuclear and Chloroplast Data Combined With Network Analyses Supports the Polyploid Origin of the Apple Tribe and the Hybrid Origin of the Maleae—Gillenieae Clade

**DOI:** 10.3389/fpls.2021.820997

**Published:** 2022-01-25

**Authors:** Richard G. J. Hodel, Elizabeth A. Zimmer, Bin-Bin Liu, Jun Wen

**Affiliations:** ^1^Department of Botany, National Museum of Natural History, Smithsonian Institution, Washington, DC, United States; ^2^State Key Laboratory of Systematic and Evolutionary Botany, Institute of Botany, Chinese Academy of Sciences, Beijing, China

**Keywords:** allopolyploidy, ancient hybridization, cytonuclear conflict, genome doubling, phylogenetic networks, phylogenomics, reticulate evolution

## Abstract

Plant biologists have debated the evolutionary origin of the apple tribe (Maleae; Rosaceae) for over a century. The “wide-hybridization hypothesis” posits that the pome-bearing members of Maleae (base chromosome number *x* = 17) resulted from a hybridization and/or allopolyploid event between progenitors of other tribes in the subfamily Amygdaloideae with *x* = 8 and *x* = 9, respectively. An alternative “spiraeoid hypothesis” proposed that the *x* = 17 of Maleae arose via the genome doubling of *x* = 9 ancestors to *x* = 18, and subsequent aneuploidy resulting in *x* = 17. We use publicly available genomic data—448 nuclear genes and complete plastomes—from 27 species representing all major tribes within the Amygdaloideae to investigate evolutionary relationships within the subfamily containing the apple tribe. Specifically, we use network analyses and multi-labeled trees to test the competing wide-hybridization and spiraeoid hypotheses. Hybridization occurred between an ancestor of the tribe Spiraeeae (*x* = 9) and an ancestor of the clade Sorbarieae (*x* = 9) + Exochordeae (*x* = 8) + Kerrieae (*x* = 9), giving rise to the clade Gillenieae (*x* = 9) + Maleae (*x* = 17). The ancestor of the Maleae + Gillenieae arose via hybridization between distantly related tribes in the Amygdaloideae (i.e., supporting the wide hybridization hypothesis). However, some evidence supports an aspect of the spiraeoid hypothesis—the ancestors involved in the hybridization event were likely both *x* = 9, so genome doubling was followed by aneuploidy to result in *x* = 17 observed in Maleae. By synthesizing existing genomic data with novel analyses, we resolve the nearly century-old mystery regarding the origin of the apple tribe. Our results also indicate that nuclear gene tree-species tree conflict and/or cytonuclear conflict are pervasive at several other nodes in subfamily Amygdaloideae of Rosaceae.

## Introduction

**FIGURE 1:**
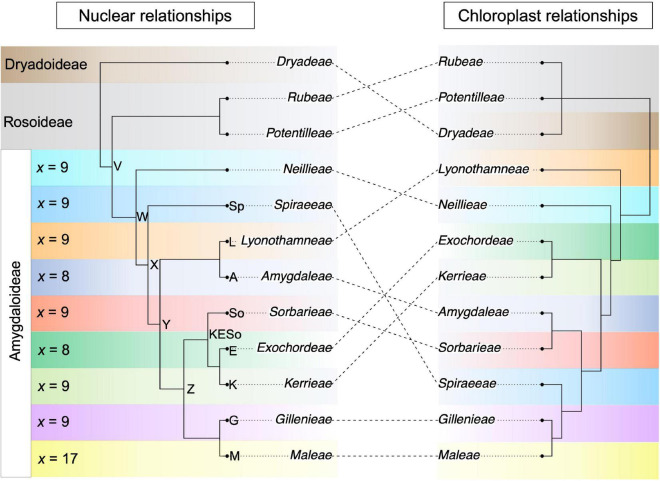
Nuclear- and chloroplast-inferred phylogenetic relationships among tribes within the Amygdaloideae, rooted using the other two subfamilies within the Rosaceae, the Dryadoideae and Rosoideae. Conflict between the nuclear and chloroplast topologies is shown using dotted lines. The tribal and subfamilial relationships are based on topologies from Xiang et al.(2017; nuclear) and Zhang et al.(2017; chloroplast). For each tribe in the Amygdaloideae, the base chromosome number is indicated to the left of the nuclear phylogeny. Note that one genus in the Maleae is not *x* = 17 but rather is *x* = 15 (*Vauquelinia*). Select nodes in the nuclear phylogeny are labeled with letters or abbreviations to facilitate reference to these nodes in the text.

Throughout the Rosaceae, there is pervasive conflict between phylogenetic relationships inferred using the nuclear vs. chloroplast genomes. Among major lineages of the Rosaceae, variation in chromosome number is prevalent, and there have been frequent whole genome duplications in the family. Many lineages of the Rosaceae contain economically important species; the Maleae, with over 1,000 species, includes commercially important fruit crops, such as apples and pears, as well as many ornamentals. In addition to apples and pears, the subfamily Amygdaloideae contains many other important species such as cherries, almonds, peaches, apricots, and plums. The branching order among the three subfamilies of the Rosaceae—Amygdaloideae, Dryadoideae, and Rosoideae—is uncertain. Nuclear data indicate that the Dryadoideae are sister to the Amygdaloideae + Rosoideae (Xiang et al., 2017), whereas phylogenetic relationships reconstructed using plastome data have still not conclusively resolved the branching order. Recent analyses inferred that the Rosoideae are sister to Amygdaloideae + Dryadoideae when using whole plastome data, or that the Amygdaloideae are sister to the Dryadoideae + Rosoideae when using whole plastomes with most ambiguous sites removed (Zhang et al., 2017). In the Amygdaloideae, the relationships between many tribes conflict when the nuclear and chloroplast topologies are compared (Figure 1; Xiang et al., 2017; Zhang et al., 2017). Furthermore, within the Rosaceae, many relationships between tribes were inconsistent between the nuclear and chloroplast genomes, such as the placement of all tribes within the Rosoideae except for Ulmarieae (Xiang et al., 2017; Zhang et al., 2017). Cytonuclear conflict also exists within the Rosaceae at shallower systematic scales (e.g., within the tribe Maleae; Liu et al., 2019, 2020a,b, 2021).

For nearly a century, plant biologists have debated the evolutionary origin of the apple tribe Maleae (Rosaceae; formerly Maloideae). Species in the tribe Maleae are characterized by a base chromosome number of *x* = 17 (except for *x* = 15 in *Vauquelinia* Corrêa ex Bonpl.)—distinct from other tribes in the Rosaceae, which typically are *x* = 7, 8, or 9 (Evans and Campbell, 2002). Within the Amygdaloideae, the subfamily containing the Maleae, all tribes except the Maleae are *x* = 8 or 9 (Robertson et al., 1991). Because the base chromosome number of Maleae was approximately double that of all its close relatives, early researchers investigated hypotheses of a polyploid origin of the pome-bearing members of the apple subtribe Malinae, which includes all Maleae except for three early diverging dry fruit lineages including genera *Kageneckia* Ruiz and Pav. (*x* = 17), *Lindleya* Kunth (*x* = 17) and *Vauquelinia* (*x* = 15) (Nebel, 1929; Campbell et al., 1995). Darlington and Moffett (1930) proposed hypotheses of autopolyploidy, which were quickly refuted by Sax (1931, 1932, 1933) after observing predominantly univalents in triploids during meiosis, as opposed to multivalents. Sax proposed an explanation of allopolyploidy occurring between *x* = 8 and *x* = 9 progenitors from the subfamily Spiraeoideae (now as part of Amygdaloideae; Potter et al., 2007). The “wide-hybridization hypothesis” formulated in the 1930s posits that the Malinae (base chromosome number *x* = 17) resulted from an ancient hybridization event between progenitors from other tribes in the subfamily Amygdaloideae that have *x* = 8 and *x* = 9, respectively. The “wide-hybridization” hypothesis was favored by Stebbins (1950) and was further supported by studies using isozymes decades later (Chevreau et al., 1985; Weeden and Lamb, 1987).

An alternative “spiraeoid hypothesis” proposed that the 17 (or in rare cases 15) chromosomes found in Maleae arose via the genome doubling of an *x* = 9 spiraeoid ancestor to *x* = 18, and subsequent aneuploidy resulting in *x* = 17 (Goldblatt, 1976; Evans and Campbell, 2002). This hypothesis is referred to as the “spiraeoid” hypothesis because the participants in allopolyploidy were considered a member of spiraeoid taxa (Goldblatt, 1976), in particular, the ancestor of the tribe Gillenieae (Evans and Campbell, 2002), which was traditionally placed in the formerly recognized subfamily Spiraeoideae (also see Gladkova, 1972). A genetic investigation of the origin of the apple tribe using one nuclear gene (Evans and Campbell, 2002) favored the spiraeoid hypothesis while rejecting the wide-hybridization hypothesis. Their study inferred that an ancestor of the tribe Gillenieae (*x* = 9), which is sister to the Maleae, experienced genome doubling and subsequent aneuploidy. Other molecular analyses of the Rosaceae did not explicitly test hypotheses explaining the origin of the apple tribe (e.g., Potter et al., 2002, 2007). To date, the two competing hypotheses have not been tested using genomic data. Recent phylogenomic studies identified pervasive cytonuclear conflict throughout the Amygdaloideae, which contains the Maleae, suggesting that ancient hybridization and/or allopolyploidization may have impacted the diversification of this group.

The time is ripe to re-evaluate these hypotheses using analyses that consider phylogenomic data from both nuclear and chloroplast genomes, and methodologies that explicitly incorporate discordance and/or reticulation into phylogenies. As researchers obtain more DNA sequence data from both the nuclear and chloroplast genomes, it is becoming increasingly clear that cytonuclear conflict is prevalent in many plant lineages (Huang et al., 2014; Bruun-Lund et al., 2017; Lee-Yaw et al., 2018; Hodel et al., 2021; Liu et al., 2021; Wang et al., 2021; Xu et al., 2021). Here, we limit our focus to studying and resolving cytonuclear conflict within the Amygdaloideae. Our objectives in this paper are to: (1) Test the competing wide hybridization and spiraeoid hypotheses, and investigate the role of genome doubling in the origin of the apple tribe using genomic data from the nuclear and chloroplast genomes, and (2) Characterize cytonuclear conflict within the Amygdaloideae, a clade with pervasive reticulate evolution, and identify explanations for the observed conflict. Specifically, we integrate data from Xiang et al. (2017)—hundreds of nuclear genes—and plastomes from Zhang et al. (2017), supplemented by chloroplast sequence data from NCBI, to investigate pervasive cytonuclear conflict within the Amygdaloideae that may provide insights into the evolutionary origin of the apple tribe.

## Materials and Methods

### Dataset Construction

Subfamily Amygdaloideae contains approximately 1,500 species organized into nine tribes (Figure 1). Some tribes, such as Maleae and Amygdaleae are represented by hundreds of species, whereas others such as Gillenieae and Lyonothamneae each contain a single genus. We selected representatives from each tribe, as well as species from the other Rosaceae subfamilies Dryadoideae and Rosoideae, with the goal of obtaining a representative sampling of Amygdaloideae tribes while limiting the number of taxa included so that certain analyses (i.e., phylogenetic networks) would be computationally feasible. First, we downloaded the 148-taxa alignments of 882 nuclear genes from Xiang et al. (2017) from TreeBASE (study ID = 19726). Briefly, Xiang et al. (2017) isolated RNA from young leaf, floral bud, or fruit tissue, performed transcriptome sequencing, and identified putative low copy candidate orthologous genes to use in phylogenentic analyses. The publicly available alignments consisted of consensus sequences from the candidate orthologous genes. As the authors of Xiang et al. (2017) note, a large proportion of these 882 nuclear genes are suspected hidden paralogs, and they used several paralog filtering steps. Xiang et al. (2017) primarily used smaller filtered subsets of genes (571, 444, 256, and 113 genes) in phylogenomic analyses. In the present study, phylogenies were first constructed using all 148 taxa to identify putative paralogous gene trees. We inferred each of 882 gene trees from the sequence alignments using RAxML v8.2.11 (Stamatakis, 2014) with the GTRGAMMA model of evolution, 20 independent ML searches, and 100 bootstrap replicates. For consistency with Xiang et al. (2017), we screened all gene trees using TreSpEx (Struck, 2014) with the *a priori* paralogy screening function with a bootstrap threshold of 95—with two masking filters—first using established ordinal relationships, and then using subfamilial relationships. The ordinal and subfamilial filters were used in Xiang et al. (2017) to remove suspected hidden paralogs, so we used this strategy for consistency. Our TreSpEx paralog trimming left 448 putative orthologs out of 882. When we investigated including paralogs in our analyses (i.e., using all 882 genes), our species tree topology did not match the dominant topology presented in Xiang et al. (2017). Therefore, we proceeded using our 448 gene set, which did match the dominant topology from Xiang et al. (2017). We trimmed taxa from the 448-gene alignments using the “pxrmt” command in *phyx* (phylogenetic tools for unix; Brown et al., 2017) to reduce the data matrix down to 27 species. We included at least one species from each of the nine tribes in the Amygdaloideae and two species each from the Rosoideae and Dryadoideae, as well as one outgroup species, *Ziziphus jujuba* Mill. (Rhamnaceae). The trimming of taxa was done to facilitate downstream analyses (i.e., network analyses implemented in SNaQ) that become computationally intractable when larger numbers of taxa (i.e., > 30) are included. Whenever possible, we selected species represented by both nuclear data (from Xiang et al., 2017) and complete plastomes (from Zhang et al., 2017).

For the species not represented by plastome data in Zhang et al. (2017), we downloaded complete plastomes from NCBI for all species except *Physocarpus opulifolius* (L.) Maxim. (Table 1), which was represented in the nuclear data from Xiang et al. (2017) but not in the plastome data from Zhang et al. (2017). For *P. opulifolius*, we downloaded RNA-Seq reads from NCBI (accession number ERR2040427; Table 1) and used FastPlast^1^ to *de novo* assemble reads into contigs. The contigs were mapped to a reference plastome [*Malus domestica* (Suckow) Borkh., accession number: MK434916.1; Table 1] to complete the assembly. Because this species was the only taxon without a complete plastome sequence, we included two additional *Physocarpus* (Cambess.) Raf. plastomes from Zhang et al. (2017), labeled by the authors as *Physocarpus sp. A* and *Physocarpus sp. B*, in our preliminary chloroplast phylogenetic analyses to verify that the phylogenetic position of our newly assembled plastome was as expected. MAFFT (Katoh and Standley, 2013) was used to align the plastomes with settings “--maxiterate 5000 --localpair --adjustdirectionaccurately.” Resulting alignments were trimmed using TrimAl with the “-automated1” heuristic. The “pxclsq” command in *phyx* was separately used to filter the alignment based on either 20, 30, 40, 50, or 60% column occupancy required. We compared the phylogenetic trees resulting from all alignments, and after determining there was no change in topology, we used the TrimAl-trimmed tree in subsequent plastome phylogenetic analyses.

**TABLE 1:** For all 27 focal species used in our study, the NCBI accession number of the plastome sequence is listed.

Species	Chloroplast accession number	Tribe
*Prunus hypoleuca*	KT766059.1	Amygdaleae
*Prunus mume*	NC_023798.1	Amygdaleae
*Prunus yedoensis*	NC_026980.1	Amygdaleae
*Cercocarpus montanus*	KY420024.1	Dryadeae
*Dryas octopetala*	KY420029.1	Dryadeae
*Oemleria cerasiformis*	KY419923.1	Exochordeae
*Prinsepia utilis*	NC_021455.1	Exochordeae
*Gillenia stipulata*	NC_045321.1	Gillenieae
*Kerria japonica*	MN418902.1	Kerrieae
*Rhodotypos scandens*	KY419951.1	Kerrieae
*Lyonothamnus floribundus*	KY420005.1	Lyonothamneae
*Amelanchier alnifolira*	NC_045314.1	Maleae
*Cydonia oblonga*	MN061993.1	Maleae
*Kageneckia oblonga*	NC_045324.1	Maleae
*Malus domestica*	MK434916.1	Maleae
*Rhaphiolepis indica*	NC_045330.1	Maleae
*Sorbus torminalis*	NC_033975.1	Maleae
*Vauquelinia californica*	MN068269.1	Maleae
*Physocarpus opulifolius*	ERR2040427	Neillieae
*Potentilla_freyniana*	MK209638.1	Potentilleae
*Rubus coreanus*	NC_042715.1	Rubeae
*Adenostoma fasciculatum*	KY387915.1	Sorbarieae
*Sorbaria sorbifolia*	MN026875.1	Sorbarieae
*Aruncus dioicus*	MW115132.1	Spiraeeae
*Holodiscus discolor*	KY420032.1	Spiraeeae
*Petrophytum caespitosum*	KY419970.1	Spiraeeae
*Ziziphus jujuba*	KU351660.1	outgroup

*For one species, Physocarpus opulifolius, a complete plastome sequence was not available, so we generated one from raw RNA-Seq data (accession number listed in this table); assembly details provided in text. Tribe membership for each species is indicated in the rightmost column.*

We assessed the phylogenetic relationships among the 27 species using RAxML and ASTRAL to ensure that the nuclear topology reflected the relationships from Xiang et al. (2017). The ML analysis was conducted in RAxML using a concatenated supermatrix of the 448 orthologues, with the GTRGAMMA model of evolution, 20 independent ML searches, and 100 bootstrap replicates. Both unpartitioned and partitioned (-q) analyses were used. The coalescent analyses were conducted in ASTRAL (Mirarab et al., 2014), a tree estimation program consistent with the coalescent, and using quartet support values to measure confidence in species relationships. The quartet support scores indicate the percentage of quartets in gene trees that are concordant with a given branch and therefore can show the amount of gene tree conflict associated with a branch. Quartet scores provide more information about uncertainty at key nodes than bootstrap scores, which can be inappropriately inflated in some phylogenomic datasets (Roycroft et al., 2020). We also used RAxML to ensure that the chloroplast relationships from Zhang et al. (2017) were recapitulated, using the GTRGAMMA model of evolution, 20 independent ML searches, and 100 bootstrap replicates. Phylogenetic trees were visualized and manipulated using IcyTree (Vaughan, 2017) and Interactive Tree of Life (Letunic and Bork, 2021). The “cophylo” function in the R package phytools (Revell, 2012) was used to visualize concordance between the nuclear and plastome phylogenies. Unless otherwise noted, all software analyses were run on the Smithsonian Institution High Performance Cluster (SI/HPC, “Hydra”).^2^

### Network Analyses

To assess if a reticulate tree (i.e., a phylogenetic network) better represented the nuclear gene tree data than a purely bifurcating tree, we used the program SNaQ, which is implemented in PhyloNetworks (Solís-Lemus et al., 2017). The phylogenomic network method SNaQ, which uses a pseudolikelihood method, explicitly accommodates hybridization by representing certain nodes as having received genetic material from two parental lineages with inheritance probabilities γ and 1-γ. The RAxML-inferred gene trees for all 448 orthologues were used as input and summarized using quartet concordance factors (i.e., the proportion of gene trees with a given quartet; Larget et al., 2010). In SNaQ, networks are optimized based on the branch lengths and inheritance probabilities in phylogenetic network space as measured by a pseudodeviance score. The pseudodeviance score represents a multiple of the network’s log-likelihood score up to a constant where the network perfectly fits the data. Lower pseudo-deviance scores always indicate a better fit, but as *h*_*max*_ increases, the pseudodeviance score always improves (Solís-Lemus and Ané, 2016). Accordingly, the rate of change in the pseudodeviance score between *h*_*max*_ values can be used to assess the optimal *h*_*max*_ (Baudry et al., 2011). We constructed networks using *h*_*max*_ values ranging from 0 to 5. For the initial optimization (*h*_*max*_ = 0), the ASTRAL tree was used as a starting network with no hybridization edges, and for subsequent *h*_*max*_ values, the optimal network estimated by the preceding lower *h*_*max*_ value was used as the starting topology. We ran 10 independent searches for each *h*_*max*_ value and the optimal number of hybridization edges was assessed by plotting *h*_*max*_ against the log-likelihood score (i.e., network score) of the optimal network for each *h*_*max*_ value.

### Conflict Analyses

The program *phyparts* (Smith et al., 2015) was used to assess gene tree conflict in the nuclear dataset. This program compares rooted gene trees with the rooted species tree to identify topologically concordant, discordant, and uninformative gene trees for each species tree node. Because rooted gene trees were necessary, fewer gene trees (440 out of 448) were available for this analysis due to the absence of the outgroup in some gene trees. We used a gene tree bootstrap support cutoff of 50% (-s 50); below this threshold gene trees were considered to be uninformative for a given node. A *phyparts* analysis using no bootstrap support cutoff was also run for comparison. The results of each *phyparts* analysis were visualized as piecharts on the phylogeny using the *phypartspiecharts.py* jupyter notebook (by Matt Johnson).^3^ Nodes of interest, as identified by network analysis and the above conflict analysis, were further investigated using the “alternative relationship test” implemented in *phyckle* (Smith et al., 2020). The alternative relationship test takes as input two or more user specified bipartitions, which are used as a constraint when running RAxML to infer every gene tree from the sequence matrices. Log-likelihood scores are calculated for each gene tree and then compared to determine which topology (i.e., between the user-inputted bipartitions) is optimal for every gene tree. The number of gene trees and/or the summed difference of log-likelihood scores between the gene trees can then used to determine support for one bipartition vs. others.

### Allopolyploidy Analyses

The software package GRAMPA (Thomas et al., 2017) was used to identify the parental lineages involved in a hybridization event leading to an allopolyploid lineage. GRAMPA makes use of multiply-labeled (MUL) trees, which are topologies in which selected species can appear twice, a common way of representing polyploid relationships when constrained by a bifurcating phylogeny. The algorithm implemented in GRAMPA uses least common ancestor reconciliation of gene trees and species trees (Goodman et al., 1979; Page, 1994) to place polyploidy events on a phylogeny. Branches of the species tree with disproportionately high numbers of gene duplications can be used to identify polyploidy events. The use of MUL-trees enables accurate inferences of allopolyploidy vs. autopolyploidy, because all subgenomes involved in allopolyploidy can be represented as descendants of different parental linages. Under scenarios of allopolyploidy, we would expect the homoeologs that result from an allopolyploidy event to be sister to different diploid taxa (Thomas et al., 2017). Using hypotheses from the literature, and guided by the SNaQ results, we tested the following hypotheses of allopolypoidy. We considered either the Maleae (i.e., node M; Figure 1) or the Gillenieae + Maleae (node G) as possible clades that were a result of allopolyploidization (“-h1” inputs). We investigated the following nodes as potential secondary parental branches (“-h2” inputs): nodes labeled A, L, Sp, S, K, E, KESo, W, X, Y, Z (Figure 1). If the wide hybridization hypothesis is supported, we would expect a node further removed from the Gillenieae + Maleae clade to be selected as the secondary parental branch (e.g., Sp). Conversely, if the spiraeoid-origin hypothesis is supported, we would expect the “-h2” node to be adjacent to a branch representing an ancestor of Gillenieae (e.g., Z).

### Hybridization Analyses

To reconcile any differences between the phylogenetic network and MUL-tree results, we used one additional approach to test for histories of hybridization in the Amygdaloideae. The program Hybrid Detector (HyDe) uses phylogenetic invariants under a coalescent model with hybridization to infer probability of hybridization of three ingroup taxa relative to an outgroup taxon (Blischak et al., 2018). In this framework, the parameter γ represents the probability that gene trees with a hybrid population sister to parent X would arise under the parental population trees, whereas 1-γ would be the probability of a hybrid population being sister to parent Y. Based on the SNaQ results and GRAMPA results, we tested several sets of taxa for histories of hybridization in HyDe. Using the SNaQ results as a guide, we tested the hybrid status of three ingroups (Maleae + Gillenieae, Spiraeeae, Sorbarieae) relative to an outgroup (Neillieae), and based on the GRAMPA results, we tested for hybridization using three ingroups (Maleae + Gillenieae, Spiraeeae, Kerrieae + Exochordeae + Sorbarieae) and the same outgroup (Neillieae). This outgroup was chosen because it was sister to all other Amygdaloideae tribes when using nuclear data (Figure 1).

## Results

### Phylogenetic Relationships

**FIGURE 2:**
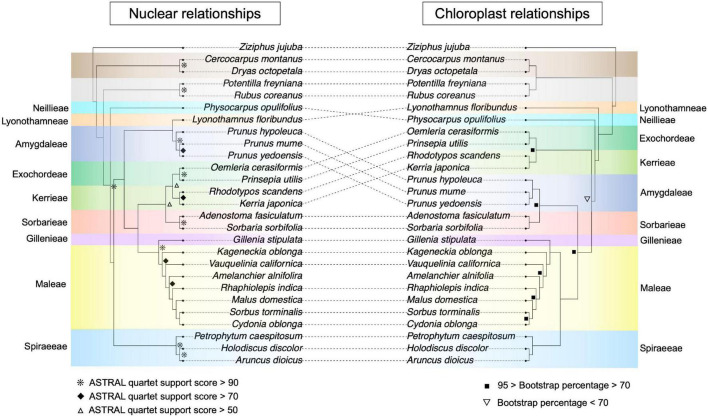
Nuclear- and chloroplast-inferred phylogenetic relationships for all 27 focal species. Tribes/subfamilies are color-coded using the same scheme as Figure 1, and tribes in the Amygdaloideae are labeled. Disagreement between the chloroplast and nuclear phylogenies is indicated by dotted lines between the phylogenies. Note that in the bottom half of the phylogenies, even though the dotted lines do not intersect, there are key differences in the branching order of clades between the two phylogenies. In the nuclear phylogeny, node support was assessed using ASTRAL quartet support scores and nodes are labeled if the quartet support was greater than 50 (hollow triangles), greater than 70 (solid diamonds), or greater than 90 (asterisks). Any unlabeled nodes in the nuclear phylogeny had quartet support scores < 50. In the chloroplast phylogeny, support was measured via bootstrapping in RAxML and nodes with bootstrap scores less than 70% are labeled (inverted hollow triangles) as are nodes with less than 95% but greater than 70% (solid squares). All unlabeled nodes in the chloroplast phylogeny have 100% bootstrap support.

Our phylogenetic analyses recovered all subfamilies and tribes as monophyletic (Figure 2). In the nuclear phylogeny, the Dryadoideae (represented by *Cercocarpus montanus* Raf. and *Dryas octopetala* L.) and Rosoideae (*Potentilla freyniana* Bornm. and *Rubus coreanus* Miq.) were successively sister to the Amygdaloideae (Figure 2). Within the Amygdaloideae, the Neillieae (*Physocarpus opulifolius*) and Spiraeeae [*Aruncus dioicus* (Walter) Fernald, *Holodiscus discolor* (Pursh) Maxim., and *Petrophytum caespitosum* (Nutt.) Rydb.] were successively sister to a clade containing the remaining seven tribes (Figure 2). The Amygdaleae [*Prunus hypoleuca* (Koehne) J.Wen, *Prunus mume* Siebold & Zucc., and *Prunus × yedoensis* Matsum.] and Lyonothamneae (*Lyonothamnus floribundus* Gray) then form a clade sister to the remaining five tribes. The Exochordeae (*Prinsepia utilis* Royle. and *Oemleria cerasiformis* (Torr. & Gray ex Hook. & Arn.) J.W.Landon), Kerrieae [*Kerria japonica* (L.) DC. and *Rhodotypos scandens* (Thunb.) Makino], and Sorbarieae [*Adenostoma fasciculatum* Hook. & Arn. and *Sorbaria sorbifolia* (L.) A.Braun] formed a clade that is sister to the clade comprised of Gillenieae [*Gillenia stipulata* (Muhl. ex Willd.) Nutt.] and Maleae [*Cydonia oblonga* Mill., *Sorbus torminalis* (L.) Crantz, *Malus domestica*, *Rhaphiolepis indica* (L.) Lindl. ex Ker Gawl., *Amelanchier alnifolia* (Nutt.) Nutt., *Vauquelinia californica* (Torr.) Sarg., and *Kageneckia oblonga* Ruiz & Pav.]. In the chloroplast phylogeny, the Dryadoideae and Rosoideae were sister to the Amygdaloideae (Figure 2). The Lyonothamneae and Neillieae were successively sister to the seven remaining tribes. Then, the Exochordeae and Kerrieae formed a clade sister to the remaining five tribes. The Amygdaleae and Sorbarieae made up a clade sister to the Spiraeeae, Gillenieae, and Maleae. Within this final clade, the Spiraeeae were sister to Maleae + Gillenieae (Figure 2).

The phylogeny constructed using nuclear data recapitulated results from Xiang et al. (2017) with our reduced-taxa dataset (Figure 2 and Supplementary Figure 1). In the nuclear datasets, there were several topological differences between the nuclear coalescent and concatenation trees (Figure 2 and Supplementary Figure 1)—differences that also existed among different datasets in Xiang et al. (2017). When comparing our nuclear phylogenies, the key difference was the placement of the Amygdaleae + Lyonothamneae clade, which was sister to the Kerrieae + Exochordeae + Sorbarieae in the concatenation trees, but in the coalescent tree was sister to these three tribes as well as the Maleae + Gillenieae (Figure 2 and Supplementary Figure 1). Within the Maleae, there were also discrepancies between the coalescent tree and concatenation trees, and between the unpartitioned and partitioned concatenation trees (Figure 2 and Supplementary Figure 1). In the coalescent topology, *Rhaphiolepis indica* and *Malus domestica* were respectively successively sister to *Cydonia oblonga* and *Sorbus torminalis* (Figure 2). However, in the unpartitioned ML phylogeny, *Malus* was sister to *Cydonia* whereas *Sorbus* was sister to *Rhaphiolepis* Lindl (Supplementary Figure 1). Meanwhile, in the partitioned ML tree, *Malus domestica* was sister to *Rhaphiolepis indica* and *Cydonia oblonga* was sister to *Sorbus torminalis* (Supplementary Figure 1). Hereafter, we use our ASTRAL topology as the nuclear topology for clarity because it matches the predominant topology presented in Xiang et al. (2017).

Our reduced-taxa plastome phylogeny matched the ML whole plastome tree topology, as opposed to the ambiguous-sites-removed tree, from Zhang et al. (2017) (Figure 2). For simplicity, we use this plastome tree in subsequent comparisons with the nuclear phylogeny, because the primary topological difference between plastome trees from Zhang et al. (2017) involved the branching order of subfamilies, not the relationships among Amygdaloideae tribes, which is our focus. As expected, there were numerous differences between our plastome and nuclear phylogenies (Figure 2) throughout the tree, including major relationships between subfamilies. In the plastome phylogeny, the Dryadoideae were sister to the Rosoideae, whereas in all nuclear trees, the Dryadoideae were sister to the Rosoideae + Amygdaloideae. There were also many differences in intertribal relationships, including virtually every tribe except the Gillenieae + Maleae (Figure 2). The different alignment strategies that we used for the plastome sequence alignment did not influence the inferred topology of the chloroplast phylogeny, but there was variation in the bootstrap percentages at certain nodes between the different alignments (Supplementary Figure 2).

### Network Analyses

**FIGURE 3:**
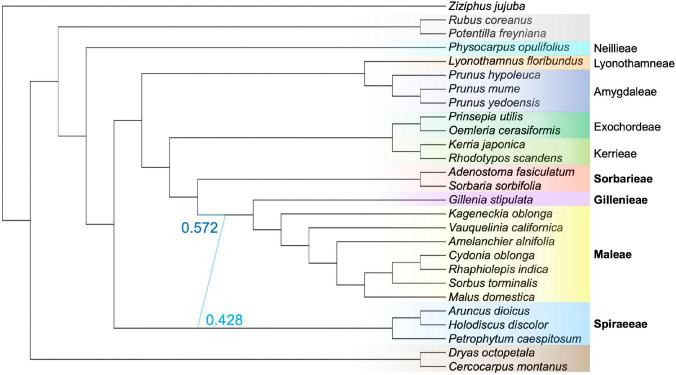
The optimal SNaQ network using 27 species with *h*_*max*_ = 1. The SNaQ analysis inferred that the lineage *Gillenia* + Maleae is 57.2% sister to an ancestor of Sorbarieae (represented here by *Adenostoma fasiculatum* and *Sorbaria sorbifolia*), and 42.8% sister to an ancestor of Spiraeeae (represented here by *Aruncus dioicus*, *Holodiscus discolor*, *Petrophytum caespitosum*). The color coding shows tribe/subfamily membership and is identical to the scheme in Figures 1, 2. Tribes in the Amygdaloideae are labeled to the right, and bold lettering indicates tribes involved in the hybridization edge.

The SNaQ network analysis inferred that one hybridization event was optimal (Figure 3 and Supplementary Figure 3). The hybridization edge indicated that the clade Gillenieae + Maleae was 57.2% sister to the Sorbarieae [represented by *Adenostoma* Hook. & Arn. and *Sorbaria* (Ser.) A.Braun], and 42.8% sister to the Spiraeeae [represented by *Aruncus* L., *Holodiscus* (K.Koch) Maxim., and *Petrophytum* (Nutt. ex Torr. & A.Gray) Rydb.; Figure 3]. The position of the Sorbarieae (57.2% sister to Gillenieae + Maleae) contrasted with both the nuclear topology (Sorbarieae sister to Exochordeae + Kerrieae) and the plastome topology (Sorbarieae sister to Amygdaleae) (Figures 2, 3). Notably, the position of the Spiraeeae as 42.8% sister to the Gillenieae + Maleae was congruent with the plastome topology, where Spiraeeae was sister to Gillenieae + Maleae. Essentially, the major hybridization edge was similar to the nuclear topology, while the minor hybridization edge was consistent with the plastome topology (Figures 2, 3). The other networks with *h*_*max*_ = 2–5 all included a hybridization edge similar to the *h*_*max*_ = 1 network (Supplementary Figure 4). As *h*_*max*_ increased, the network score always improved, although the very small changes in network score as *h*_*max*_ increases from 1 to 5 indicated that *h*_*max*_ = 1 was indeed the optimal network. Nevertheless, the hybrid edges in other networks can still provide valuable insights. The SNaQ network with *h*_*max*_ = 2 showed that the second hybridization edge was between *Prunus hypoleuca* of the *Maddenia* group (formerly in the genus *Maddenia* Hook. f & Thomson; Wen and Shi, 2012) and the lineage ancestral to Lyonothamneae + Amygdaleae (Supplementary Figure 4). This hybridization edge indicated that *Prunus hypoleuca* is 87.9% sister to the other *Prunus* L. species, and 12.1% sister to the ancestor of Lyonothamneae + Amygdaleae.

### Conflict Analyses

**FIGURE 4:**
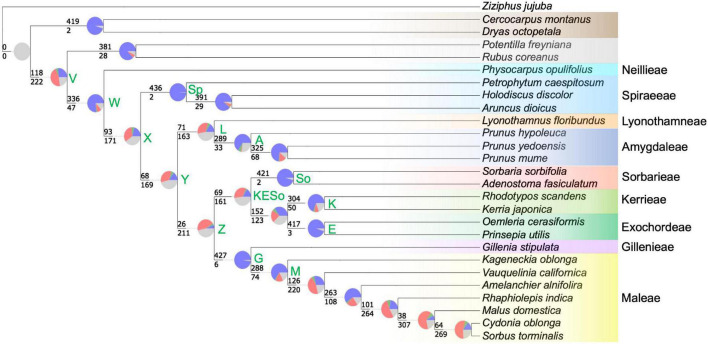
The *phyparts* tree indicating conflict at each node of the nuclear phylogeny using the 448-gene alignment and requiring gene trees to have 50% bootstrap support to be considered informative at a given node. Because *phyparts* uses rooted gene trees, only 440 genes were included in this analysis. At each node, the pie charts indicate the proportion of homologs supporting the clade defined by the node is shown in blue, the proportion supporting the primary alternative for that clade are green, the proportion supporting all other alternatives for the clade are red, and the proportion of homologs with less than 50% bootstrap support are shown in gray. Along each branch, the top number shows the number of genes concordant with the species tree at the associated node, and the bottom number represents the number of genes discordant with the species tree for the clade of interest. The color-coding of species names indicates tribe/subfamily membership and is consistent with Figures 1–3. As in Figure 1, select nodes are labeled with letters or abbreviations to enable easy reference in the text. Amygdaloideae tribes are labeled to the right of the species names.

The *phyparts* analysis indicated a wide range of gene tree conflict relative to the species tree, from virtually no conflict (e.g., the node defining the Spiraeeae; node Sp1 in Figure 4), to pervasive conflict where nearly 10 times more genes were discordant with the species tree topology than were concordant (e.g., node Z—the node defining Gillenieae + Maleae as sister to Exochordeae + Kerrieae + Sorbarieae; Figure 4). The nodes with a greater proportion of gene trees in conflict with the species tree than congruent with the species tree generally reflected nodes which disagree between the nuclear and chloroplast phylogenies, even though the data used for this analysis were nuclear gene trees and the nuclear species tree. The nodes with high conflict included deep nodes such as those displaying uncertainty regarding subfamilial relationships (node V; Figure 4) and the one reflecting the uncertainty of the position of the Spiraeeae tribe relative to the other tribes of the Amygdaloideae (node X; Figure 4). Moreover, the sister relationship between Amygdaleae + Lyonothamneae and a clade comprised of five other tribes (Sorbarieae, Kerrieae, Exochordeae, Gillenieae, and Maleae) showed high gene tree/species tree conflict (node Y; Figure 4). One other relatively deep node, representing the clade Kerrieae + Exochordeae + Sorbarieae (node KESo; Figure 4) exhibited high gene tree/species tree conflict, with over twice as many gene trees discordant as concordant. There were also several nodes with high degrees of discord within the Maleae, but investigating these shallower relationships is beyond the scope of this study, and we focused our taxon sampling with the goal of investigating deeper relationships in the tree as opposed to investigating documented discordance within the Maleae. When no bootstrap cutoff was used to consider whether gene trees were informative for a given node, the results were qualitatively similar (Supplementary Figure 5), so we focused on reporting the proportions of gene trees using the 50% bootstrap threshold (Figure 4). Based on the results of the SNaQ analysis, we used the *phyckle* “alternative relationship test” to further investigate support for the placement of the Spiraeeae using nuclear genes. We found that over 45% of nuclear genes (203 out of 448) support the chloroplast topology over the nuclear topology regarding the placement of Spiraeeae (Table 2). Moreover, the sum of log-likelihood differences across all genes indicated greater gene tree support for the chloroplast topology than the nuclear topology (Table 2).

**TABLE 2:** The results of the *phyckle* analysis investigating gene tree support for alternative topologies regarding the phylogenetic placement of the tribe Spiraeeae.

Conflict	Topology	Bipartition	Number genes	Sum lnL difference
	nuclear	(*Aruncus, Holodiscus, Petrophytum, Cercocarpus, Dryas, Potentilla, Rubus, Ziziphus, Physocarpus*) | (all other taxa)	245	2503.2
Phylogenetic position of Spiraeeae				
	chloroplast	(*Aruncus, Holodiscus, Petrophytum, Sorbus, Vauquelinia, Amelanchier, Cydonia, Gillenia, Kageneckia, Malus, Rhaphiolepis*) | (all other taxa)	203	3115.8

*For the chloroplast and nuclear topologies, the conflicting bipartitions, the number of genes supporting each relationship and the sum of log-likelihood differences for genes supporting each bipartition are shown.*

### Multiply-Labeled Tree Analysis

**FIGURE 5:**
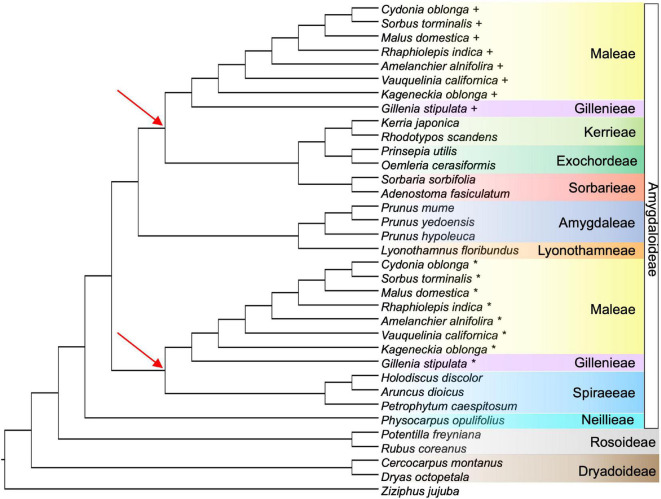
The most parsimonious tree from the GRAMPA analysis, which is a multi-labeled (MUL) tree indicating two tips for all species in Maleae + Gillenieae. For each species with multiple labels, the first tip is indicated by a plus sign and the second tip is shown using an asterisk. One Gillenieae + Maleae clade is sister to Kerrieae + Exochordeae + Sorbarieae, and the other Gillenieae + Maleae clade is sister to Spiraeeae. The red arrows highlight the nodes defining different lineages sister to the multi-labeled taxa in Maleae + Gillenieae. The color-coding of species shows tribe/subfamily and is consistent with all previous figures.

The GRAMPA analysis revealed that an allopolyploid event likely occurred in the clade that resulted in Gillenieae + Maleae. The most parsimonious tree (score = 14,733) was a MUL-tree with multiple tips of all taxa within the Gillenieae + Maleae, with one clade sister to Exochordeae + Kerrieae + Sorbarieae, and one clade sister to the Spiraeeae (Figure 5). This MUL-tree was more parsimonious than the singly labeled tree (score = 14,777), which is considered evidence of allopolyploidy. The result that the Spiraeeae are one parental participant in an allopolyploidy event was consistent with the SNaQ network results. One difference between the most parsimonious GRAMPA MUL-tree and the optimal SNaQ network was that the GRAMPA tree shows Exochordeae + Kerrieae + Sorbarieae as sister to the Gillenieae + Maleae, whereas in the SNaQ network, an ancestor of the Sorbarieae was one half of the hybridization edge (Figures 3, 5).

### Hybridization Analyses

Hybrid Detector analyses confirmed aspects of both the SNaQ and GRAMPA results (Table 3). While using the Neillieae as an outgroup, the HyDe analysis inferred that the clade Maleae + Gillenieae was a hybrid with parents Spiraeeae and Sorbarieae, confirming the phylogenetic network result, and rejecting the possibility that either parental lineage (i.e., Spiraeeae or Sorbarieae) could be the hybrid lineage in this case (Table 3). The γ-value from the test that showed Maleae + Gillenieae as a hybrid lineage was 0.262 (Table 3). A similar analysis to test the relationship found using GRAMPA recovered support for Maleae + Gillenieae as a hybrid lineage with parents Spiraeeae and Kerrieae + Exochordeae + Sorbarieae (Table 3). Here the γ-value when Maleae + Gillenieae were a hybrid lineage was 0.526 (Table 3).

**TABLE 3:** The two hybridization hypotheses tested using Hybrid Detector.

	Parent 1	Hybrid	Parent 2	γ-value	Z-score	*P*-value
Hybrid relationship inferred by SNaQ	Sorbarieae	Maleae-Gillenieae	Spiraeeae	0.262	14.321	0.000
	Sorbarieae	Spiraeeae	Maleae-Gillenieae	–1.233	–99999.9	1.000
	Spiraeeae	Sorbarieae	Maleae-Gillenieae	0.608	–25.934	1.000

Hybrid relationship inferred by GRAMPA	Spiraeeae	Maleae-Gillenieae	Kerrieae-Exochordeae-Sorbarieae	0.526	21.569	0.000
	Spiraeeae	Kerrieae-Exochordeae-Sorbarieae	Maleae-Gillenieae	0.911	–2.349	0.991
	Maleae-Gillenieae	Spiraeeae	Kerrieae-Exochordeae-Sorbarieae	–0.122	–2.118	0.983

*The first HyDe analysis (top) found support for the hypothesis of Maleae-Gillenieae as a hybrid taxon resulting from parents Sorbarieae and Spiraeeae, which is consistent with the SNaQ result. The second HyDe analysis (bottom) inferred that Maleae-Gillenieae was a hybrid of parents Spiraeeae and Kerrieae-Exochordeae-Sorbarieae, which corresponds to the GRAMPA results.*

## Discussion

The important role of hybridization and genome doubling in generating plant diversity is becoming apparent (Soltis and Soltis, 2009; Folk et al., 2018). However, there are few well-supported examples of large, successful groups such as Maleae originating via wide hybridization and/or allopolyploidy from the ancestor of a small lineage (i.e., *Gillenia* Moench) (Evans and Campbell, 2002). Based on several complementary analyses—the comparison of nuclear and chloroplast phylogenies, phylogenetic network analyses, and allopolyploidy analyses using MUL-trees—we test the competing wide hybridization and spiraeoid hypotheses, and investigate the role of genome doubling, to explain the origin of the apple tribe. Here, we present multiple lines of evidence indicating that an ancestor of the Spiraeeae was likely the maternal participant in an ancient hybridization event and an ancestor of the clade Sorbarieae + Exochordeae + Kerrieae was likely the paternal participant, although there was some minor variation in analyses regarding the identity of the paternal parent (Figures 3, 5). This hybridization event likely explains the origin of the clade Gillenieae + Maleae (Figure 3). Our results indicate that aspects of both existing hypotheses explaining the origin of the apple tribe are correct, but also aspects of each were incorrect. Our results also indicate that nuclear gene tree-species tree conflict and/or cytonuclear conflict are pervasive at several nodes in the Amygdaloideae. This suggests that beyond the hybrid origin of the apple clade, other lineages in the Amygdaloideae have reticulate evolutionary histories characterized by hybridization and/or allopolyploidy. Below, we discuss the details of our results and their implications on the origin of the apple tribe, as well as the possible explanations for high conflict nodes elsewhere in the subfamily Amygdaloideae.

### The Ancient Hybrid Origin of Maleae-Gillenieae and Subsequent Genome Doubling in Maleae

Our results suggest the ancestor of the Maleae + Gillenieae originated via hybridization between distantly related tribes in the Amygdaloideae (i.e., the wide hybridization hypothesis, which states that the Maleae are the result of an ancient hybridization event between progenitors from other tribes in the subfamily Amygdaloideae) (Figure 3). Specifically, there was a hybridization event between an ancestor of the tribe Spiraeeae (*x* = 9) and an ancestor of Sorbarieae (*x* = 9) + Exochordeae (*x* = 8) + Kerrieae (*x* = 9), which gave rise to the clade comprised of Gillenieae (*x* = 9) + Maleae (*x* = 17) (Figure 3). This result is largely congruent with the wide hybridization hypothesis, except that we found that the clade Gillenieae + Maleae was the result of a wide hybridization event, as opposed to just the Maleae (Figure 3). Our results also partially support the spiraeoid hypothesis (i.e., the 17 chromosomes found in Maleae arose via the genome doubling of an *x* = 9 ancestor to *x* = 18, and subsequent aneuploidy resulting in *x* = 17), specifically regarding the role of whole genome duplication in the origin of the Maleae (Figure 5). The ancestors involved in the hybridization event leading to Gillenieae + Maleae had base chromosome numbers of *x* = 8 or 9, so there may have been genome doubling, possibly followed by aneuploidy if two *x* = 9 taxa were involved, to result in the *x* = 17 observed in the Maleae (Figure 5). Regardless of the ancestral chromosome number (*x* = 8 vs. *x* = 9), the genome doubling aspect of the spiraeoid hypothesis is supported by our results. However, given that our network analysis found that the clade Gillenieae + Maleae was the result of a hybridization event, and the base chromosome number of Gillenieae is *x* = 9, a genome doubling event preceding Gillenieae + Maleae can readily explain the *x* = 17 observed in the Maleae but not *x* = 9 in Gillenieae (Figure 3). The Gillenieae lineage may have undergone diploidization following an allopolyploidy event whereas the Maleae did not. Interpretation of the GRAMPA analysis favors this explanation because the most parsimonious allopolyploidy event precedes Gillenieae + Maleae, as opposed to only Maleae. Alternatively, perhaps there was a second genome doubling event of Maleae after an initial hybridization event leading to Gillenieae + Maleae. We favor the latter explanation, which is consistent with our SNaQ analysis and with results from Xiang et al. (2017), who noted nodes in the Maleae with evidence of whole genome duplications (WGDs) after the divergence of the Maleae from the ancestor of Gillenieae + Maleae (see Xiang et al., 2017; Figure 5). The annotated genome assembly of another Gillenieae species, *Gillenia trifoliata*, revealed that many syntenic blocks in *Gillenia trifoliata* mapped to two locations in *Malus domestica*, as would be expected with a history of genome doubling (Ireland et al., 2021). Moreover, the same syntenic blocks correspond to single orthologous regions in other Rosaceae species [*Rubus occidentalis* (raspberry) of Rosoideae and *Prunus persica* (peach)] of Amygdaloideae, suggesting that it is unlikely that the Gillenieae underwent a WGD and subsequent diploidization—a simpler explanation is that the WGD occurred after an initial hybridization leading to Gillenieae + Maleae.

Across the plant tree of life, diversification via genome duplication is relatively common. It is becoming increasingly clear that following WGD events, the genomes of organisms are particularly malleable and that genomic rearrangements may spur key functional innovations. Genome evolution associated with WGDs has often been studied in crop species, many of which are polyploid. For example, controlled crosses of early generation allopolyploid wheat revealed that aneuploidy is common following WGDs (Zhang et al., 2013). However, there are examples of variation in genome size and organization after WGD events in non-model systems. In the neopolyploid *Tragopogon* L., massive chromosomal variation followed an allopolyploidy event (Chester et al., 2012), including aneuploidy in 69% of cases. Within a single genus of ca. 250 species (clover; genus *Trifolium* L.), there have been many deviations from the ancestral chromosome state (2*n* = 16), including at least 22 instances of polyploidy and 19 occurrences of aneuploidy (Ellison et al., 2006). The diversification of the Gillenieae-Maleae clade may represent another example of lineages that diversified following chromosomal rearragnements via allopolyploidy and aneuploidy.

Many previous studies have hypothesized an allopolyploid origin of the apple tribe (Sax, 1931, 1932, 1933; Stebbins, 1950; Chevreau et al., 1985; Weeden and Lamb, 1987; Robertson et al., 1991; Evans and Campbell, 2002; Vamosi and Dickinson, 2006; Potter et al., 2007). The wide-hybridization hypothesis, favored until 2002, considered many lineages as possible participants in hybridization and/or allopolyploidy, but strong evidence for any particular lineage was lacking. The spiraeoid hypothesis was supported by one duplicated nuclear gene (*GBSSI-1* and *GBSSI-2*; Evans and Campbell, 2002), inferring that both parental participants in allopolyploidy were ancestors of the Gillenieae lineage. Our network analyses (Figure 3) indicate that a hybridization event between an ancestor of the Spiraeeae and Sorbarieae leading to Gillenieae + Maleae, whereas allopolyploid analyses (Figure 5) indicate that an ancestor of the Spiraeeae and a common ancestor of Sorbarieae + Exochordeae + Kerrieae were likely the parental participants in allopolyploidy. Both of these scenarios were confirmed as possible hybridization events using separate analyses (i.e., HyDe; Table 3). Given the low support for the KESo and Z nodes (quartet support scores = 50.44 and 39.70, respectively, and high degrees of gene tree conflict; Figure 4), perhaps the topological uncertainty in the nuclear phylogeny is causing the discrepancy between the SNaQ and GRAMPA analyses (Figures 3, 5). When considering the bifurcating nuclear and plastome topologies (Figure 2), and considering the proportions of nuclear gene trees that support the nuclear vs. chloroplast topologies (Table 2), it becomes evident that the Spiraeeae ancestor was most likely the maternal donor to a hybridization or allopolyploid event because Spiraeeae is sister to Gillenieae + Maleae in the plastome tree, and that the ancestor of Sorbarieae + Exochordeae + Kerrieae was the paternal participant because this relationship is more similar to the nuclear tree than the plastome tree.

### Discordance/Reticulation Throughout the Amygdaloideae

There are multiple nodes with pervasive conflict, both among the subfamilies of the Rosaceae and within the Amygdaloideae. These include nodes V (Rosoideae—Amygdaloideae sister), Y (Lyonothamneae + Amygdaleae sister to clade defined by node Z), L (Lyonothamneae sister to Amygdaleae), Z (Kerrieae + Exochordeae + Sorbarieae sister to Gillenieae +Maleae), and KESo (Sorbarieae sister to Kerrieae + Maleae) (Figure 4). Xiang et al. (2017) produced six distinct Rosaceae phylogenies based on data filtering (between 113 and 882 genes included) and tree-inference method (concatenation with ML inference in RAxML vs. a coalescent species tree approach implemented in ASTRAL). They defined nodes as highly supported (100% bootstrap support in all trees), moderately supported (90% bootstrap support in at least five trees, and 85% support in all six trees), poorly supported (80% bootstrap support in three or more trees and 40% support in all six trees), and unresolved (not meeting the above criteria). Multiple nodes with pervasive conflict according to our *phyparts* analysis were considered highly supported (Y,V,L) or moderately supported (KESo) in Xiang et al. (2017). Only one key node with high gene tree conflict (Z) was considered poorly supported in Xiang et al. (2017), and no nodes with pervasive conflict identified in our analyses were listed as unresolved. None of the above nodes were consistent with the plastome tree, either from Zhang et al. (2017) or from our analyses. Clearly, there is substantial conflict among nuclear gene trees within the Amygdaloideae, in addition to the documented cytonuclear discord. Histories of reticulate evolution appear common in this group, beyond the allopolyploid origin of the apple tribe.

We cannot be certain of the cause of conflict in many of the nodes in the Amygdaloideae. Potential biological explanations for gene tree discord may include incomplete lineage sorting (ILS) or hybridization. Processes such as ILS may also lead to gene tree-species tree conflict in the absence of hybridization. However, there is strong evidence for the hybrid origin of the Gillenieae + Maleae. SNaQ is robust to ILS in that it can incorporate uncertainty in user-estimated gene trees and handle gene tree discordance caused by ILS (Solís-Lemus and Ané, 2016). The comparison of pseudodeviance network scores in SNaQ between the ASTRAL tree, which accommodates ILS, and the *h*_*max*_ = 1 network, which can accommodate ILS and hybrid edges, clearly favors the *h*_*max*_ = 1 network. The *phyckle* analysis of the node defining the position of the Spiraeeae (i.e., node X, Figure 4 and Table 2) on its own can identify the proportion of nuclear genes that support species tree or alternative relationships, but does not explicitly identify sources of conflict. However, the nearly equal distribution of nuclear genes that support the nuclear topology and the chloroplast topology, when considered alongside the other analyses (e.g., SNaQ, GRAMPA, and HyDe), add evidence that a history of hybridization via allopolyploidy shaped evolutionary histories of the sampled genes. While we do not have specific expectations for the proportion of gene trees that may conflict with the species tree solely due to ILS, that so many gene trees support the alternative chloroplast topology, as opposed to a distribution of different topologies induced by ILS, provides more evidence for an instance of hybridization. That over 45% of nuclear genes (203 out of 448) support the chloroplast topology over the nuclear topology with regard to the placement of Spiraeeae is another piece of evidence that the maternal participant in allopolyploidy leading to the apple tribe was an ancestor of the Spiraeeae. The large number of nuclear genes that favor the chloroplast topology may in part explain past uncertainty in phylogenetic studies investigating the Rosaceae or its subfamilies.

The tribe Lyonothamneae is represented by a monotypic genus, *Lyonothamnus* A.Gray. The position of this tribe varies greatly between the plastome phylogeny (Lyonothamneae sister to all other tribes in the Amygdaloideae) and nuclear phylogeny (Lyonothamneae sister to Amygdaleae). Furthermore, there is substantial nuclear gene tree conflict at this node (L; Figure 4). The SNaQ network with *h*_*max*_ = 2 showed that the second hybridization edge was between *Prunus hypoleuca* of the *Maddenia* group and the lineage ancestral to Lyonothamneae + Amygdaleae (Supplementary Figure 4). Essentially, this hybridization edge means that *Prunus hypoleuca* of the *Maddenia* group (Wen and Shi, 2012) is 87.9% sister to the other *Prunus* species, and 12.1% sister to the ancestor of Lyonothamneae + Amygdaleae. The interpretation of this hybridization edge is less straightforward than the *h*_*max*_ = 1 edge. However, there is evidence from previous studies that a WGD occurred near the base of the Amygdaleae (Xiang et al., 2017), and other studies have hypothesized that ancient hybridization and/or allopolyploidy were involved in the diversification of *Prunus* (Chin et al., 2014; Zhao et al., 2016, 2018; Hodel et al., 2021), the sole accepted genus in the Amygdaleae. Future studies with denser taxon-sampling in the Amygdaleae and hundreds of nuclear loci combined with chloroplast data are needed to investigate the evolutionary history of the Lyonothamneae + Amygdaleae.

Although assessing discordance within the Maleae is not a focus of this paper, we note that in the *h*_*max*_ = 4 and *h*_*max*_ = 5 SNaQ networks (Supplementary Figure 4), there are hybridization edges that indicate possible hybridization within the Maleae. The *h*_*max*_ = 4 hybrid edge shows *Kageneckia* 96.2% sister to all other Maleae but also 3.8% sister to Maleae + Gillenieae. In the *h*_*max*_ = 5 network, the hybrid edge indicates the ancestor of subtribe Malinae (pome-bearing Maleae, i.e., Maleae excluding *Kageneckia* and *Vauquelinia*) is 97.7% sister to *Vauquelinia* and also 2.3% sister to Maleae + Gillenieae. Taken in isolation, these hybrid edges mean little, especially given the discrepancy between the γ values of the major and minor hybridization edges. However, when considered in concert with previously documented discord within the Maleae, the conflict documented via *phyparts* at multiple nodes in the Maleae (Figure 4), as well as evidence of genome doubling at multiple nodes within the Maleae (Xiang et al., 2017), the *h*_*max*_ = 4 and *h*_*max*_ = 5 SNaQ results point to hybridization, especially introgression, as a possible mechanism explaining phylogenomic discord within the Maleae. Further targeted investigations are needed to address discordance within the Maleae.

The well-documented discordance between chloroplast and nuclear phylogenies in the Amygdaloideae could also be explained by chloroplast capture. This phenomenon occurs when native cytoplasm is replaced by foreign cytoplasm via hybridization followed by repeated backcrossing (Rieseberg and Soltis, 1991). In closely related species that are sexually compatible, chloroplast capture can be pervasive and lead to cytonuclear discordance. In the Amygdaloideae, there have been several instances of chloroplast capture documented. In the Amygdaleae tribe, cytonuclear discord was attributed to chloroplast capture in several *Prunus* species, including North American plums (Rohrer et al., 2008) and East Asian cherries (Cho et al., 2014). Within the Maleae, there is also evidence of chloroplast capture as a mechanism causing cytonuclear discord. Strong discordance between nuclear and plastid phylogenies regarding the placement of the Maleae genera *Malacomeles* (Decne.) Decne. and *Peraphyllum* Nutt. supports ancient chloroplast capture events in SW North America (Liu et al., 2020a). Because chloroplast capture involves hybridization followed by recurrent backcrossing, it occurs more frequently at shallower systematic scales among sexually compatible species. Accordingly, chloroplast capture could explain the SNaQ hybridization edges detected within the Maleae and Amygdaleae at higher values of *h*_*max*_ (Supplementary Figure 4). Additionally, although we did not detect cytonuclear discord within the Maleae in our sampling, histories consistent with chloroplast capture may explain the pervasive gene tree conflict at nodes within the Maleae (Figure 4).

### Synthesizing Multiple Nuclear Genes and Chloroplast Data Resolves Cases of Reticulation

Previous molecular studies of the Rosaceae typically used either nuclear (e.g., Evans and Campbell, 2002) or chloroplast data (Potter et al., 2002). The single-nuclear gene *GBSSI* phylogeny by Evans and Campbell (2002) could not resolve the position of the Spiraeeae, and the branching order of the Spiraeeae, Sorbarieae, Exochordeae, Amygdaleae, and Dryadeae was a polytomy. Potter et al. (2002) used two chloroplast genes and recovered a phylogeny that placed the Spiraeeae + Sorbarieae sister to the Gillenieae + Maleae + Amygdaleae. However, there was poor bootstrap support (i.e., < 75%) for all these relationships except Gillenieae + Maleae. Potter et al.’s (2002) chloroplast phylogeny also found that Lyonothamneae were sister to all other Amygdaloid tribes with 100% bootstrap support. One study that used data from both nuclear and chloroplast genomes is Potter et al. (2007), with six nuclear and four chloroplast loci to create a consensus phylogeny, inferred that the Spiraeeae were sister to the Gillenieae + Maleae (i.e., the dominant chloroplast topology from Zhang et al. (2017) and the present study), albeit with low support (44% bootstrap and 57% Bayesian clade credibility). Potter et al. (2007) excluded two nuclear loci from their analyses due to results “inconsistent in some ways with the majority of other data.” Two of the anomalous results caused by the two excluded nuclear genes they report are inconsistent placement of the Spiraeeae and the lack of a sister relationship between Lyonothamneae and the rest of the Amygdaloideae (referred to as Spiraeoideae in Potter et al., 2007).

Subsequent results, from Xiang et al. (2017) and Zhang et al. (2017) and the present study, contextualize and explain the results from earlier molecular studies. The position of Lyonothamneae is clearly quite different in the chloroplast and nuclear genomes, and this is reflected throughout the literature; studies with only chloroplast data repeatedly find Lyonothamneae sister to the rest of Amygdaloideae, typically with strong support. In contrast, this relationship is never found in studies using only nuclear data. The position of the Spiraeeae has been variable in studies from the literature, but it is now becoming clear that much of the uncertainty with regard to its placement is due to a history of WGDs in the Amygdaloideae. Specifically, in this paper we characterize one instance of allopolyploidy, in which an ancestor of the Spiraeeae was likely the maternal participant in allopolyploidization. Given the distinct chloroplast and nuclear topologies regarding the placement of Spiraeeae, and the fact that nearly half of nuclear genes sampled in this study favor the chloroplast topology, it is unsurprising that earlier molecular studies using fewer than 10 markers were unable to confidently resolve the position of Spiraeeae. Although we used a set of complementary analyses to resolve the origin of the apple tribe, there is clearly more phylogenetic uncertainty due to reticulate evolutionary histories in the Amygdaloideae. The different positions of the Lyonothamneae in the nuclear and chloroplast phylogenies, coupled with the SNaQ network results and previous evidence of WGD events leading to and within the Amygdaleae, indicate that future targeted efforts should be focused on resolving the evolutionary history of the Lyonothamneae and Amygdaleae.

## Conclusion and Prospects

Over the past several decades, systematists have embraced the need for incorporating genealogical information from nuclear genes to obtain robust estimates of phylogeny. Chloroplast data were favored for many years due to their high copy number which translated to easy generation of homologous loci for many individuals and/or species (Thode et al., 2020; Wang et al., 2020; Welker et al., 2020). Those studies fell out of favor due to the limited information regarding ancestry given their typical uniparental inheritance. It is now becoming clear that reticulation is prevalent at many phylogenetic scales due to hybridization or other processes (Lee-Yaw et al., 2018). In cases of pervasive reticulation, nuclear AND chloroplast data are now necessary complements to one another if researchers hope to resolve reticulate complexes. Our study highlights how synthesizing results from existing studies cannot only reconcile differences from two recent studies, but also answer century old questions that have been continually debated in the literature. Our study also highlights the need to revisit and reconsider phylogenetic relationships, even when they have been found to be highly supported using metrics such as bootstrapping. In a number of recent studies (e.g., Soltis et al., 2004; Prasanna et al., 2020; Walker et al., 2021), careful analyses of conflict have revealed that we should not be overly confident in apparently resolved relationships. In conclusion, our results from multiple lines of evidence confirmed the hybrid origin of the Maleae + Gillenieae clade and supported the polyploidy-aneuploidy-origin aspect of the hypothesis of Maleae (*x* = 17 or 15) originating from the tribe Gillenieae (*x* = 9) as proposed by Evans and Campbell (2002). Future research may provide a complete picture of the role of hybridization in the early diversification of Maleae, especially regarding the formation of the chromosome number of 15 in *Vauquelinia* and the evolutionary mechanisms leading from dry fruits (capsules) to fleshy fruits (pomes).

## Data Availability Statement

The original contributions presented in the study are included in the article/Supplementary Material, further inquiries can be directed to the corresponding author/s.

## Author Contributions

RH, EZ, and JW conceptualized the project. RH and B-BL obtained and analyzed the data. RH led the writing of the manuscript. All authors edited drafts and approved the final version of the manuscript.

## Conflict of Interest

The authors declare that the research was conducted in the absence of any commercial or financial relationships that could be construed as a potential conflict of interest.

## Publisher’s Note

All claims expressed in this article are solely those of the authors and do not necessarily represent those of their affiliated organizations, or those of the publisher, the editors and the reviewers. Any product that may be evaluated in this article, or claim that may be made by its manufacturer, is not guaranteed or endorsed by the publisher.

## Footnotes

1
https://github.com/mrmckain/Fast-Plast.git

2
https://doi.org/10.25572/SIHPC

3
https://github.com/mossmatters/MJPythonNotebooks/blob/master/phypartspiecharts.py

## References

[B1] Baudry J.-P. Maugis C. Michel B. (2011). Slope heuristics: overview and implementation. *Stat. Comput.* 22 455–470. 10.1007/s11222-011-9236-1

[B2] Blischak P. D. Chifman J. Wolfe A. D. Kubatko L. S. (2018). HyDe: a python package for genome-scale hybridization detection. *Syst. Biol.* 67 821–829. 10.1093/sysbio/syy023 29562307 PMC6454532

[B3] Brown J. W. Walker J. F. Smith S. A. (2017). Phyx: phylogenetic tools for unix. *Bioinformatics* 33 1886–1888. 10.1093/bioinformatics/btx063 28174903 PMC5870855

[B4] Bruun-Lund S. Clement W. L. Kjellberg F. Rønsted N. (2017). First plastid phylogenomic study reveals potential cyto-nuclear discordance in the evolutionary history of *Ficus* L. (*Moraceae*). *Mol. Phylogenet. Evol.* 109 93–104. 10.1016/j.ympev.2016.12.031 28042043

[B5] Campbell C. S. Donoghue M. J. Baldwin B. G. Wojciechowski M. F. (1995). Phylogenetic relationships in *Maloideae* (Rosaceae): evidence from sequences of the internal transcribed spacers of nuclear ribosomal DNA and its congruence with morphology. *Am. J. Bot.* 82 903–918. 10.1002/j.1537-2197.1995.tb15707.x

[B6] Chester M. Gallagher J. P. Symonds V. V. Da Silva A. V. C. Mavrodiev E. V. Leitch A. R. (2012). Extensive chromosomal variation in a recently formed natural allopolyploid species, *Tragopogon miscellus* (Asteraceae). *Proc. Natl. Acad. Sci. U.S.A.* 109 1176–1181. 10.1073/pnas.1112041109 22228301 PMC3268322

[B7] Chevreau E. Lespinasse Y. Gallet M. (1985). Inheritance of pollen enzymes and polyploid origin of apple (*Malus x domestica* Borkh.). *Theor. Appl. Genet.* 71 268–277. 10.1007/BF00252066 24247393

[B8] Chin S.-W. Shaw J. Haberle R. Wen J. Potter D. (2014). Diversification of almonds, peaches, plums and cherries – molecular systematics and biogeographic history of *Prunus* (Rosaceae). *Mol. Phylogenet. Evol.* 76 34–48. 10.1016/j.ympev.2014.02.024 24631854

[B9] Cho M. S. Kim C. S. Kim S. H. Kim T. O. Heo K. I. Jun J. (2014). Molecular and morphological data reveal hybrid origin of wild *Prunus yedoensis* (Rosaceae) from Jeju Island, Korea: implications for the origin of the flowering cherry. *Am. J. Bot.* 101 1976–1986. 10.3732/ajb.1400318 25366862

[B10] Darlington C. D. Moffett A. A. (1930). Primary and secondary chromosome balance in *Pyrus*. *J. Genet.* 22 129–151. 10.1007/bf02983843

[B11] Ellison N. W. Liston A. Steiner J. J. Williams W. M. Taylor N. L. (2006). Molecular phylogenetics of the clover genus (*Trifolium-Leguminosae*). *Mol. Phylogenet. Evol.* 39 688–705. 10.1016/j.ympev.2006.01.004 16483799

[B12] Evans R. C. Campbell C. S. (2002). The origin of the apple subfamily (Maloideae; Rosaceae) is clarified by DNA sequence data from duplicated *GBSSI* genes. *Am. J. Bot.* 89 1478–1484. 10.3732/ajb.89.9.1478 21665749

[B13] Folk R. A. Soltis P. S. Soltis D. E. Guralnick R. (2018). New prospects in the detection and comparative analysis of hybridization in the tree of life. *Am. J. Bot.* 105 364–375. 10.1002/ajb2.1018 29683488

[B14] Gladkova V. N. (1972). On the origin of subfamily Maloideae. *Bot. Zhur.* 57 42–49.

[B15] Goldblatt P. (1976). Cytotaxonomic studies in the tribe Quillajeae (Rosaceae). *Ann. Miss. Bot. Gard.* 63 200–206. 10.2307/2395226

[B16] Goodman M. Czelusniak J. William Moore G. Romero-Herrera A. E. Matsuda G. (1979). Fitting the gene lineage into its species lineage, a parsimony strategy illustrated by cladograms constructed from globin sequences. *Syst. Biol.* 28, 132–163. 10.2307/2412519

[B17] Hodel R. G. J. Zimmer E. Wen J. (2021). A phylogenomic approach resolves the backbone of *Prunus* (Rosaceae) and identifies signals of hybridization and allopolyploidy. *Mol. Phylogenet. Evol.* 160:107118. 10.1016/j.ympev.2021.107118 33609711

[B18] Huang D. I Hefer C. A. Kolosova N. Douglas C. J. Cronk Q. C. B. (2014). Whole plastome sequencing reveals deep plastid divergence and cytonuclear discordance between closely related balsam poplars, *Populus balsamifera* and *P. trichocarpa* (Salicaceae). *New Phytol.* 204 693–703. 10.1111/nph.12956 25078531

[B19] Ireland H. S. Wu C. Deng C. H. Hilario E. Saei A. Erasmuson S. (2021). The *Gillenia trifoliata* genome reveals dynamics correlated with growth and reproduction in Rosaceae. *Hortic. Res.* 8:233. 10.1038/s41438-021-00662-4 34719690 PMC8558331

[B20] Katoh K. Standley D. M. (2013). Mafft multiple sequence alignment software version 7: improvements in performance and usability. *Mol. Biol. Evol.* 30 772–780. 10.1093/molbev/mst010 23329690 PMC3603318

[B21] Larget B. R. Kotha S. K. Dewey C. N. Ané C. (2010). Bucky: gene tree/species tree reconciliation with Bayesian concordance analysis. *Bioinformatics* 26 2910–2911. 10.1093/bioinformatics/btq539 20861028

[B22] Lee-Yaw J. A. Grassa C. J. Joly S. Andrew R. L. Rieseberg L. H. (2018). An evaluation of alternative explanations for widespread cytonuclear discordance in annual sunflowers (*Helianthus*). *New Phytol.* 221 515–526. 10.1111/nph.15386 30136727

[B23] Letunic I. Bork P. (2021). Interactive tree of life (iTOL) v5: an online tool for phylogenetic tree display and annotation. *Nucleic Acids Res.* 49 W293–W296. 10.1093/nar/gkab301 33885785 PMC8265157

[B24] Liu B.-B. Campbell C. S. Hong D. Y. Wen J. (2020a). Phylogenetic relationships and chloroplast capture in the *Amelanchier-Malacomeles-Peraphyllum* clade (Maleae, Rosaceae): evidence from chloroplast genome and nuclear ribosomal DNA data using genome skimming. *Mol. Phylogenet. Evol.* 147:106784. 10.1016/j.ympev.2020.106784 32135308

[B25] Liu B.-B. Hong D.-Y. Zhou S. L. Xu C. Dong W. P. Johnson G. (2019). Phylogenomic analyses of the *Photinia* complex support the recognition of a new genus *Phippsiomeles* and the resurrection of a redefined *Stranvaesia* in Maleae (Rosaceae). *J. Syst. Evol.* 57 678–694. 10.1111/jse.12542

[B26] Liu B.-B. Liu G.-N. Hong D.-Y. Wen J. (2020b). *Eriobotrya* belongs to *Rhaphiolepis* (Maleae, Rosaceae): evidence from chloroplast genome and nuclear ribosomal DNA data. *Front. Plant Sci.* 10:1731. 10.3389/fpls.2019.01731 32117331 PMC7019104

[B27] Liu B.-B. Ren C. Kwak M. Hodel R. G. J. Xu C. He J. (2021). Phylogenomic analyses in the apple genus *Malus* s.l. reveal widespread hybridization and allopolyploidy driving the diversifications, with insights into the complex biogeographic history in the Northern Hemisphere. *bioRxiv* [Preprint] 10.1101/2021.10.12.464085 35274452

[B28] Mirarab S. Reaz R. Bayzid M. S. (2014) ASTRAL: genome-scale coalescent-based species tree estimation. *Bioinformatics* 30, i541–i548. 25161245 10.1093/bioinformatics/btu462 PMC4147915

[B29] Nebel B. (1929). Zur cytologie von malus und vitis. *Die Gart. Bauwiss.* 1 549–592.

[B30] Page R. D. M. (1994). Maps between trees and cladistic analysis of historical associations among genes, organisms, and areas. *Syst. Biol.* 43, 58–77. 10.1093/sysbio/43.1.58

[B31] Potter D. Eriksson T. Evans R. C. Oh S. Smedmark J. E. E. Morgan D. R. (2007). Phylogeny and classification of Rosaceae. *Plant Syst. Evol.* 266 5–43.

[B32] Potter D. Gao F. Bortiri P. E. Oh S.-H. Baggett S. (2002). Phylogenetic relationships in Rosaceae inferred from chloroplast *matK* and *trnL-trnF* nucleotide sequence data. *Plant Syst. Evol.* 231 77–89.

[B33] Prasanna A. N. Gerber D. Kijpornyongpan T. Aime M. C. Doyle V. P. Nagy L. G. (2020). Model choice, missing data, and taxon sampling impact phylogenomic inference of deep basidiomycota relationships. *Syst. Biol.* 69 17–37. 10.1093/sysbio/syz029 31062852 PMC7115942

[B34] Revell L. J. (2012). phytools: an R package for phylogenetic comparative biology (and other things). *Methods Ecol. Evol.* 3 217–223. 10.1111/j.2041-210x.2011.00169.x

[B35] Rieseberg L. H. Soltis D. E. (1991). Phylogenetic consequences of cytoplasmic gene flow in plants. *Evol. Trends Plants* 5 65–84.

[B36] Robertson K. R. Phipps J. B. Rohrer J. R. Smith P. G. (1991). A synopsis of genera in Maloideae (Rosaceae). *Syst. Bot.* 16:376. 10.2307/2419287

[B37] Rohrer J. R. O’brien M. A. Anderson J. A. (2008). Phylogenetic analysis of North American plums (*Prunus* sect. *Prunocerasus*: Rosaceae) based on nuclear *Leafy* and *s6pdh* sequences. *J. Bot. Res. Inst. Tx* 2 401–414.

[B38] Roycroft E. J. Moussalli A. Rowe K. C. (2020). Phylogenomics uncovers confidence and conflict in the rapid radiation of Australo-Papuan rodents. *Syst. Biol.* 69 431–444. 10.1093/sysbio/syz044 31225616

[B39] Sax K. (1931). The origin and relationships of the Pomoideae. *J. Arnold Arbor.* 12 3–22.

[B40] Sax K. (1932). Chromosome relationships in Pomoideae. *J. Arnold Arbor.* 13 363–367.

[B41] Sax K. (1933). The origin of the Pomoideae. *Proc. Am. Soc. Hortic. Sci.* 30 147–150. 10.1046/j.1365-2672.1997.00377.x 12455904

[B42] Smith S. A. Moore M. J. Brown J. W. Yang Y. (2015). Analysis of phylogenomic datasets reveals conflict, concordance, and gene duplications with examples from animals and plants. *BMC Evol. Biol.* 15:150. 10.1186/s12862-015-0423-0 26239519 PMC4524127

[B43] Smith S. A. Walker-Hale N. Walker J. F. Brown J. W. (2020). Phylogenetic conflicts, combinability, and deep phylogenomics in plants. *Syst. Biol.* 69 579–592. 10.1093/sysbio/syz078 31747023

[B44] Solís-Lemus C. Ané C. (2016). Inferring phylogenetic networks with maximum pseudolikelihood under incomplete lineage sorting. *PLoS Genet.* 12:e1005896. 10.1371/journal.pgen.1005896 26950302 PMC4780787

[B45] Solís-Lemus C. Bastide P. Ané C. (2017). PhyloNetworks: a package for phylogenetic networks. *Mol. Biol. Evol.* 34 3292–3298. 10.1093/molbev/msx235 28961984

[B46] Soltis D. E. Albert V. A. Savolainen V. Hilu K. Qiu Y. L. Chase M. W. (2004). Genome-scale data, angiosperm relationships, and “ending incongruence”: a cautionary tale in phylogenetics. *Trends Plant Sci.* 9 477–483. 10.1016/j.tplants.2004.08.008 15465682

[B47] Soltis P. S. Soltis D. E. (2009). The role of hybridization in plant speciation. *Annu. Rev. Plant Biol.* 60 561–588. 10.1146/annurev.arplant.043008.092039 19575590

[B48] Stamatakis A. (2014). Raxml version 8: a tool for phylogenetic analysis and post-analysis of large phylogenies. *Bioinformatics* 30 1312–1313. 10.1093/bioinformatics/btu033 24451623 PMC3998144

[B49] Stebbins G. L. (1950). *Variation and Evolution in Plants.* New York, NY: Columbia University Press.

[B50] Struck T. H. (2014). Trespex-detection of misleading signal in phylogenetic reconstructions based on tree information. *Evol. Bioinformat.* 10 51–67. 10.4137/EBO.S14239 24701118 PMC3972080

[B51] Thode V. A. Lohmann L. G. Sanmartín I. (2020). Evaluating character partitioning and molecular models in plastid phylogenomics at low taxonomic levels: a case study using *Amphilophium* (Bignonieae, Bignoniaceae). *J. Syst. Evol.* 58 1071–1089.

[B52] Thomas G. W. C. Ather S. H. Hahn M. W. (2017). Gene-tree reconciliation with MUL-trees to resolve polyploidy events. *Syst. Biol.* 66 1007–1018. 10.1093/sysbio/syx044 28419377

[B53] Vamosi J. C. Dickinson T. A. (2006). Polyploidy and diversification: a phylogenetic investigation in Rosaceae. *Int. J. Plant Sci.* 167 349–358.

[B54] Vaughan T. G. (2017). IcyTree: rapid browser-based visualization for phylogenetic trees and networks. *Bioinformatics* 33 2392–2394. 10.1093/bioinformatics/btx155 28407035 PMC5860111

[B55] Walker J. F. Smith S. A. Hodel R. G. J. Moyroud E. (2021). Concordance-based approaches for the inference of relationships and molecular rates with phylogenomic data sets. *Syst. Biol.* 7:syab052. 10.1093/sysbio/syab052 34240209

[B56] Wang H. X. Morales-Briones D. F. Moore M. J. Wen J. Wang H. F. (2021). A phylogenomic perspective on gene tree conflict and character evolution in Caprifoliaceae using target enrichment data, with Zabelioideae recognized as a new subfamily. *J. Syst. Evol.* 59 897–914.

[B57] Wang Y.-B. Liu B.-B. Nie Z.-L. Chen H.-F. Chen F.-J. Figlar R. B. (2020). Major clades and a revised classification of *Magnolia* and *Magnoliaceae* based on whole plastid genome sequences via genome skimming. *J. Syst. Evol.* 58 673–695. 10.1111/jse.12588

[B58] Weeden N. Lamb R. (1987). Genetics and linkage analysis of 19 isozyme loci in apple. *J. Am. Soc. Hortic. Sci.* 112 865–872.

[B59] Welker C. A. D. Mckain M. R. Estep M. C. Pasquet R. S. Chipabika G. Pallangyo B. (2020). Phylogenomics enables biogeographic analysis and a new subtribal classification of the Andropogoneae (Poaceae—Panicoideae). *J. Syst. Evol.* 58:10031030.

[B60] Wen J. Shi W. (2012). Revision of the *Maddenia* clade of *Prunus* (Rosaceae). *PhytoKeys* 11 39–59. 10.3897/phytokeys.11.2825 22577333 PMC3332034

[B61] Xiang Y. Huang C. H. Hu Y. Wen J. Li S. Yi T. (2017). Evolution of Rosaceae fruit types based on nuclear phylogeny in the context of geological times and genome duplication. *Mol. Biol. Evol.* 34 262–281. 27856652 10.1093/molbev/msw242 PMC5400374

[B62] Xu L.-L. Yu R.-M. Lin X.-R. Zhang B.-W. Li N. Lin K. (2021). Different rates of pollen and seed gene flow cause branch-length and geographic cytonuclear discordance within Asian butternuts. *New Phytol.* 232 388–403. 10.1111/nph.17564 34143496 PMC8519134

[B63] Zhang H. Bian Y. Gou X. Zhu B. Xu C. Qi B. (2013). Persistent whole-chromosome aneuploidy is generally associated with nascent allohexaploid wheat. *Proc. Natl. Acad. Sci. U.S. A.* 110 3447–3452. 10.1073/pnas.1300153110 23401544 PMC3587266

[B64] Zhang S. D. Jin J. J. Chen S. Y. Chase M. W. Soltis D. E. Li H. T. (2017). Diversification of Rosaceae since the late *Cretaceous* based on plastid phylogenomics. *New Phytol.* 214 1355–1367. 10.1111/nph.14461 28186635

[B65] Zhao L. Jiang X.-W. Zuo Y. Liu X.-L. Chin S.-W. Haberle R. (2016). Multiple events of allopolyploidy in the evolution of the racemose lineages in *Prunus* (Rosaceae) based on integrated evidence from nuclear and plastid data. *PLoS One* 11:e0157123. 10.1371/journal.pone.0157123 27294529 PMC4905661

[B66] Zhao L. Potter D. Xu Y. Liu P. L. Johnson G. Chang Z. Y. (2018). Phylogeny and spatio-temporal diversification of *Prunus* subgenus *Laurocerasus* section *Mesopygeum* (Rosaceae) in the Malesian region. *J. Syst. Evol.* 56 637–651. 10.1111/jse.12467

