# Supplementary material for: Synthesis of Nuclear and Chloroplast Data Combined With Network Analyses Supports the Polyploid Origin of the Apple Tribe and the Hybrid Origin of the Maleae—Gillenieae Clade
Source: Front Plant Sci. 2022 Jan 25;12:820997. doi: 10.3389/fpls.2021.820997 (PMC8822239; doi:10.3389/fpls.2021.820997)
Supplement: Supplementary file 1 [file Data_Sheet_1.pdf]

Supplementary Material:

Synthesis of nuclear and chloroplast data combined with network analyses supports the polyploid origin of the apple tribe and the hybrid origin of the Maleae – Gillenieae clade

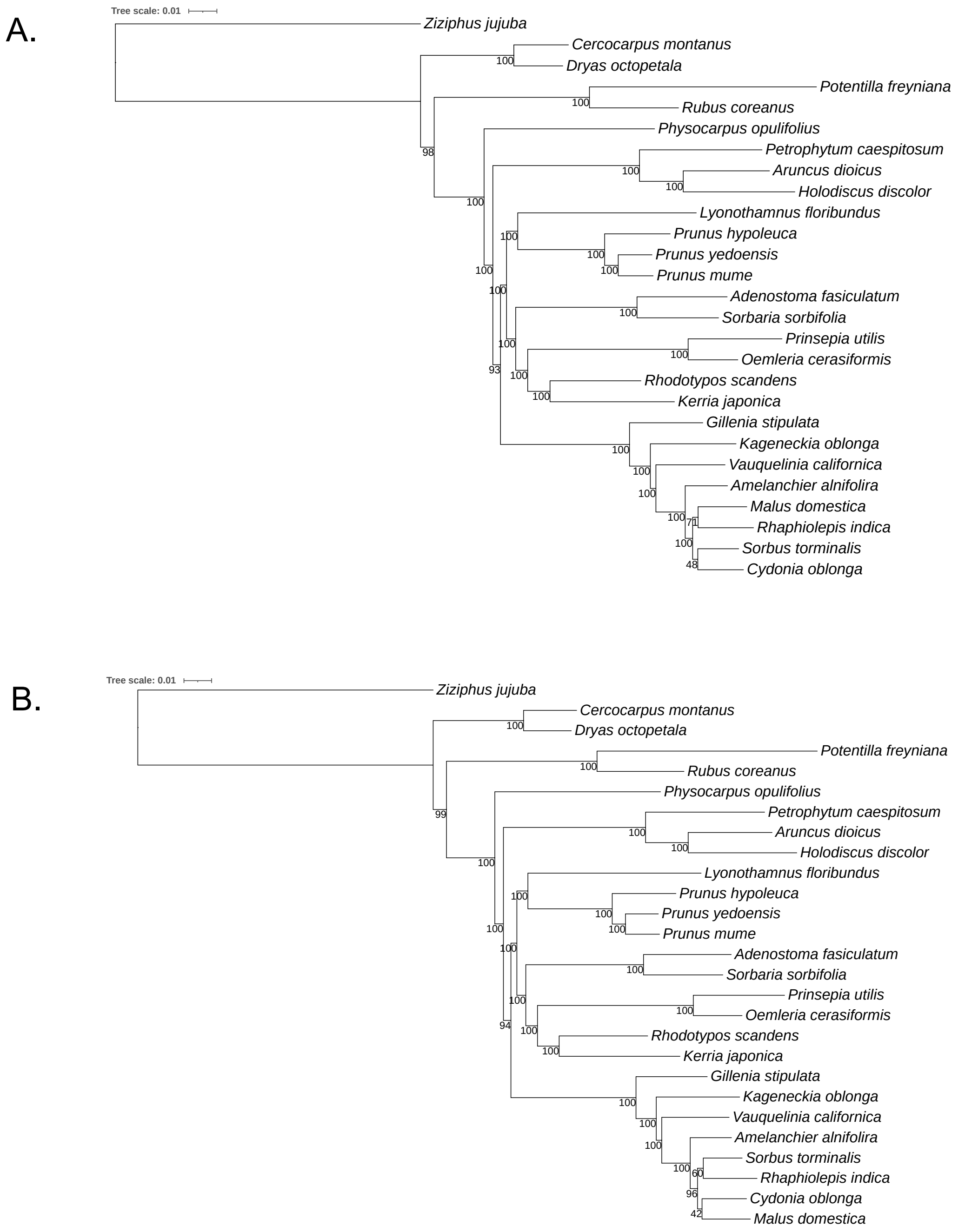

Supplemental Figure 1. The maximum likelihood nuclear phylogenies inferred using a concatenation approach using RAxML with sequence alignments either **A)** partitioned by locus or **B)** unpartitioned. The values at nodes are bootstrap percentages and the scale bar shows nucleotide substitutions per site. There are minor topological differences between these two phylogenies, all within the Maleae. There are differences between these concatenation phylogenies and the coalescent species tree phylogeny, discussed in the text.

A.

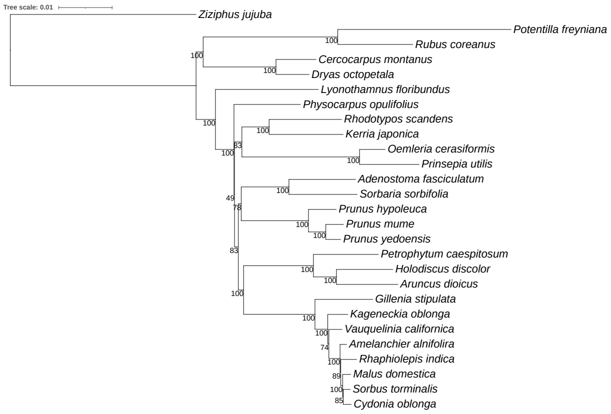
Tree scale: 0.01
Ziziphus jujuba
Potentilla freyniana
Rubus coreanus
Cercocarpus montanus
Dryas octopetala
Lyonothamnus floribundus
Physocarpus opulifolius
Rhodotypos scandens
Kerria japonica
Oemleria cerasiformis
Prinsepia utilis
Adenostoma fasciculatum
Sorbaria sorbifolia
Prunus hypoleuca
Prunus mume
Prunus yedoensis
Petrophytum caespitosum
Holodiscus discolor
Aruncus dioicus
Gillenia stipulata
Kageneckia oblonga
Vauquelinia californica
Amelanchier alnifolira
Rhaphiolepis indica
Malus domestica
Sorbus torminalis
Cydonia oblonga

B.

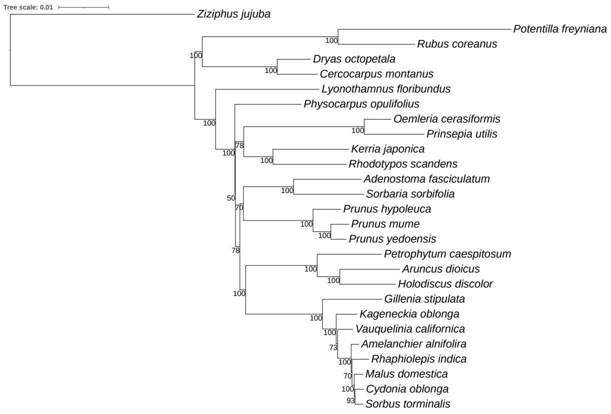
Tree scale: 0.01
Ziziphus jujuba
Potentilla freyniana
Rubus coreanus
Dryas octopetala
Cercocarpus montanus
Lyonothamnus floribundus
Physocarpus opulifolius
Oemleria cerasiformis
Prinsepia utilis
Kerria japonica
Rhodotypos scandens
Adenostoma fasciculatum
Sorbaria sorbifolia
Prunus hypoleuca
Prunus mume
Prunus yedoensis
Petrophytum caespitosum
Aruncus dioicus
Holodiscus discolor
Gillenia stipulata
Kageneckia oblonga
Vauquelinia californica
Amelanchier alnifolira
Rhaphiolepis indica
Malus domestica
Cydonia oblonga
Sorbus torminalis

C.

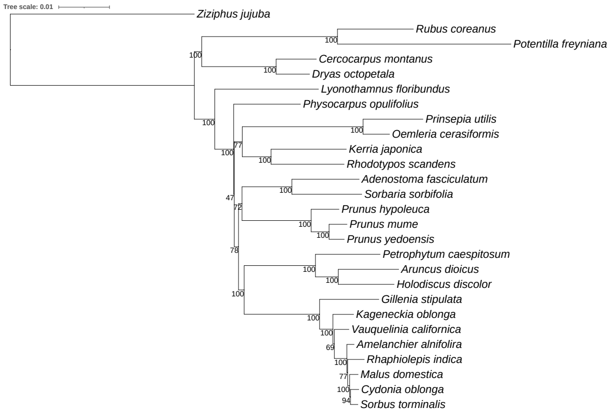
Tree scale: 0.01
Ziziphus jujuba
Rubus coreanus
Potentilla freyniana
Cercocarpus montanus
Dryas octopetala
Lyonothamnus floribundus
Physocarpus opulifolius
Prinsepia utilis
Oemleria cerasiformis
Kerria japonica
Rhodotypos scandens
Adenostoma fasciculatum
Sorbaria sorbifolia
Prunus hypoleuca
Prunus mume
Prunus yedoensis
Petrophytum caespitosum
Aruncus dioicus
Holodiscus discolor
Gillenia stipulata
Kageneckia oblonga
Vauquelinia californica
Amelanchier alnifolira
Rhaphiolepis indica
Malus domestica
Cydonia oblonga
Sorbus torminalis

D.

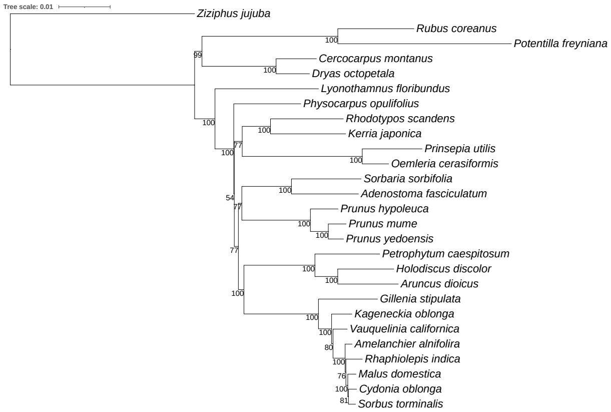

E.

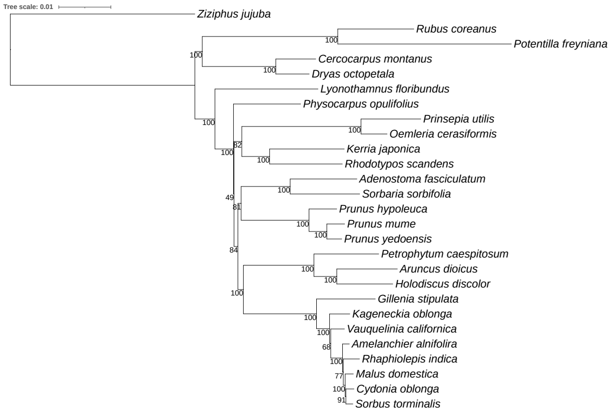

F.

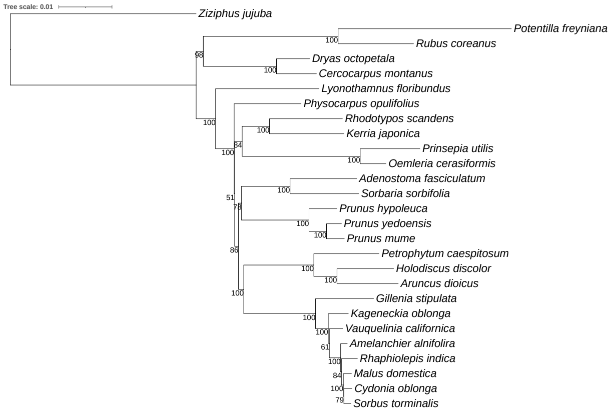

Supplemental Figure 2. Plastome phylogenies inferred using a concatenated maximum likelihood approach in RAxML. The six phylogenies are based on different alignment strategies: **A)** using TrimAl, **B)** using pxclsq with 0.2 minimum site occupancy, **C)** pxclsq with 0.3, **D)** pxclsq with 0.4, **E)** pxclsq with 0.5, **F)** pxclsq with 0.4. The topologies of all plastome trees are the same but there is some variation in bootstrap percentage scores.

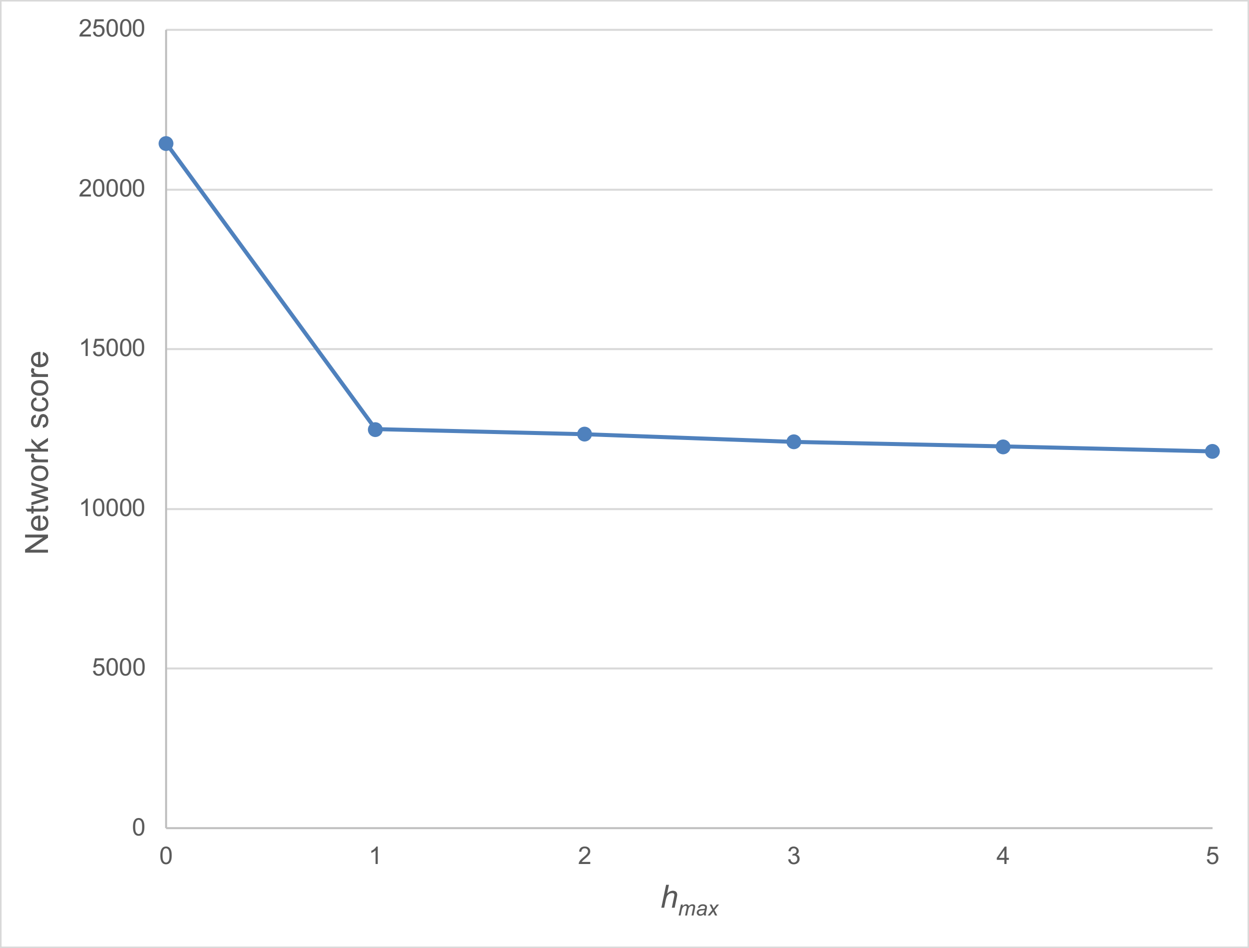

Supplemental Figure 3. For h_{max} values 0 through 5, the pseudolikelihood network score from the SNaQ analysis. The $h_{max} = 0$ tree was the purely bifurcating ASTRAL species tree and subsequent networks were allowed h_{max} hybridization edges. The network with one hybridization edge is considered optimal because there was a large change in the network score between $h_{max} = 0$ and $h_{max} = 1$ relative to any other change as h_{max} increased.

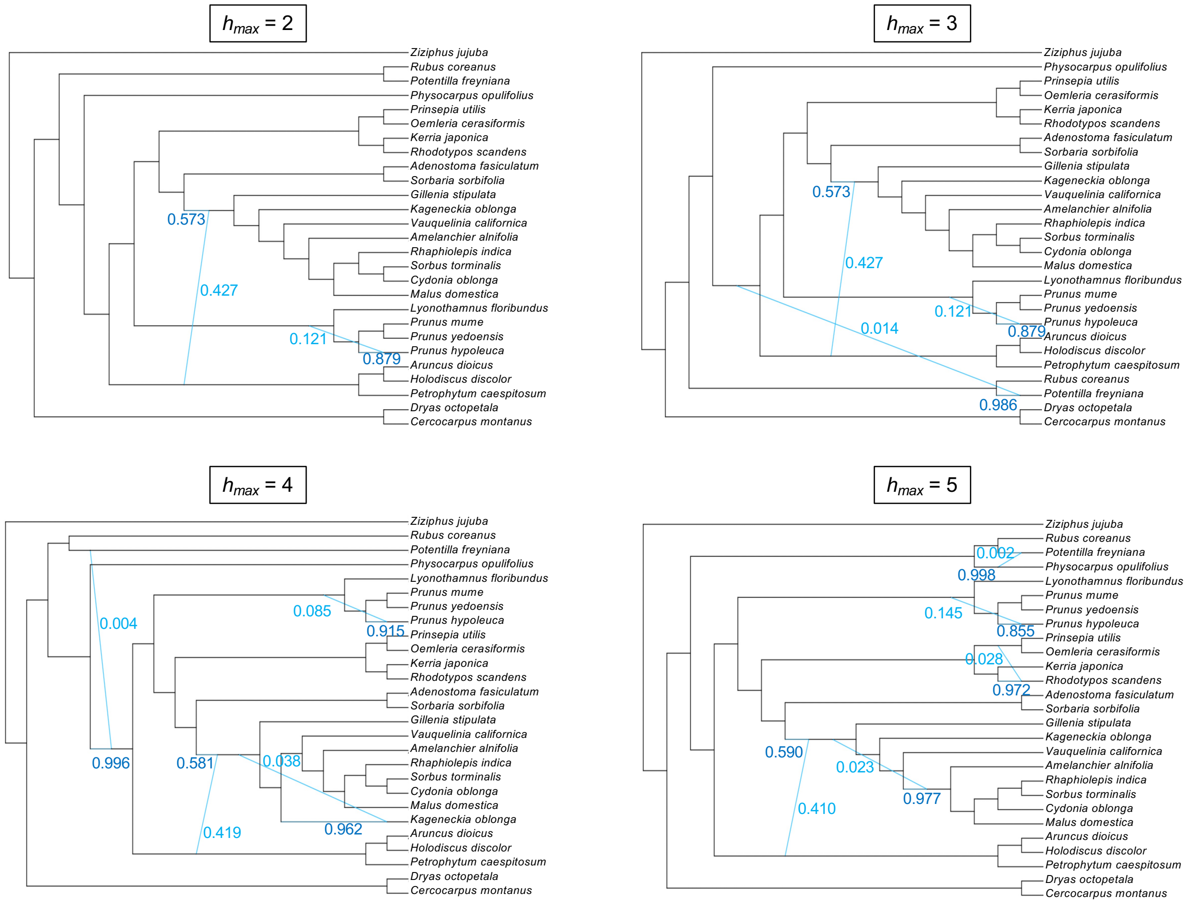

Supplemental Figure 4. The SNaQ networks for h_{max} = 2 through h_{max} = 5. Although the h_{max} = 1 network was considered optimal, networks with larger h_{max} values can still provide valuable information, especially in clades with histories of multiple reticulations.

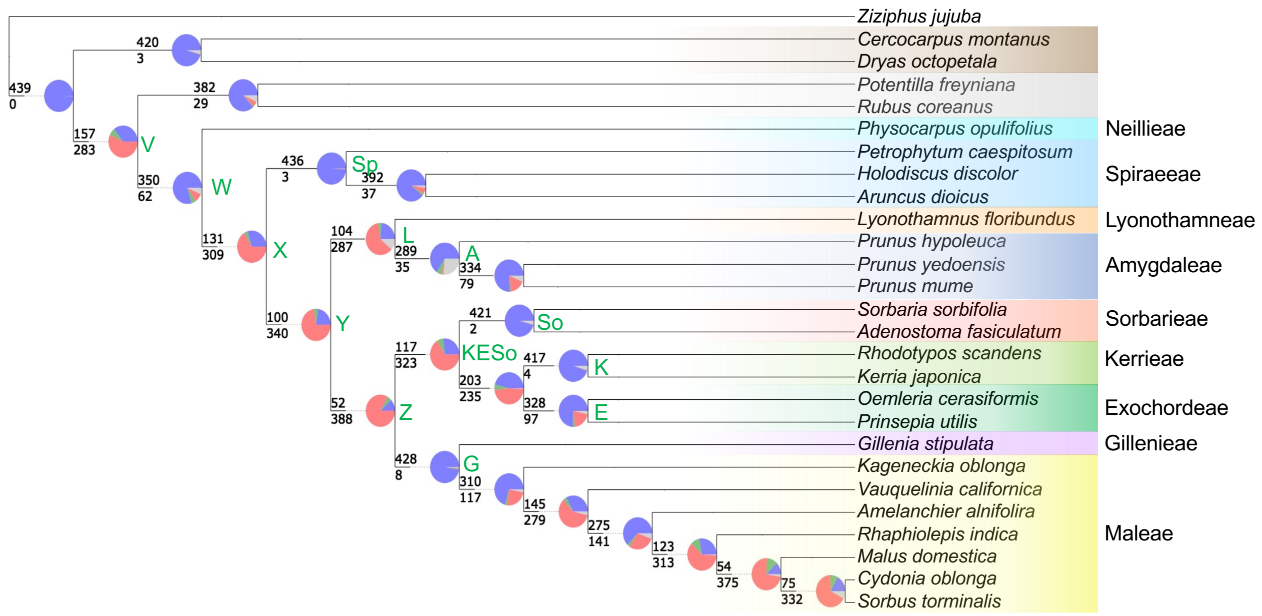

Supplemental Figure 5. The *phyparts* tree indicating conflict at each node of the nuclear phylogeny using the 448-gene alignment without requiring gene trees to have any level of bootstrap support to be considered informative at a given node. Because *phyparts* uses rooted gene trees, only 440 genes were included in this analysis. At each node, the pie charts indicate the proportion of homologs supporting the clade defined by the node is shown in blue, the proportion supporting the primary alternative for that clade are green, the proportion supporting all other alternatives for the clade are red, and the proportion of homologs with less than 50% bootstrap support are shown in gray. Along each branch, the top number shows the number of genes concordant with the species tree at the associated node, and the bottom number represents the number of genes discordant with the species tree for the clade of interest. The color-coding of species names indicates tribe/subfamily membership and is consistent with Figs. 1-5. Select nodes are labeled with letters or abbreviations to enable easy reference in the text. Amygdaloideae tribes are labeled to the right of the species names.
